# Mitochondria as target to inhibit proliferation and induce apoptosis of cancer cells: the effects of doxycycline and gemcitabine

**DOI:** 10.1038/s41598-020-61381-9

**Published:** 2020-03-09

**Authors:** Sas N. Dijk, Margherita Protasoni, Marilena Elpidorou, Albert M. Kroon, Jan-Willem Taanman

**Affiliations:** 1 Research Department of Surgical Biotechnology, Division of Surgery and Interventional Science, University College London, London, NW3 2PF UK; 2 Department of Clinical and Movement Neurosciences, Queen Square Institute of Neurology, University College London, London, NW3 2PF UK

**Keywords:** Biochemistry, Cancer, Cell biology, Oncology

## Abstract

Doxycycline has anti-tumour effects in a range of tumour systems. The aims of this study were to define the role mitochondria play in this process and examine the potential of doxycycline in combination with gemcitabine. We studied the adenocarcinoma cell line A549, its mitochondrial DNA-less derivative A549 ρ° and cultured fibroblasts. Treatment with doxycycline for 5 days resulted in a decrease of mitochondrial-encoded proteins, respiration and membrane potential, and an increase of reactive oxygen species in A549 cells and fibroblasts, but fibroblasts were less affected. Doxycycline slowed proliferation of A549 cells by 35%. Cellular ATP levels did not change. Doxycycline alone had no effect on apoptosis; however, in combination with gemcitabine given during the last 2 days of treatment, doxycycline increased caspase 9 and 3/7 activities, resulting in a further decrease of surviving A549 cells by 59% and of fibroblasts by 24% compared to gemcitabine treatment alone. A549 ρ° cells were not affected by doxycycline. Key effects of doxycycline observed in A549 cells, such as the decrease of mitochondrial-encoded proteins and surviving cells were also seen in the cancer cell lines COLO357 and HT29. Our results suggest that doxycycline suppresses cancer cell proliferation and primes cells for apoptosis by gemcitabine.

## Introduction

In the 1920s, Warburg’s ground-breaking studies on cancer metabolism revealed that tumour cells exhibit an increased flux of glucose to lactate compared to normal cells, even under aerobic conditions^1^. This so-called Warburg effect is often misconstrued as evidence of a mitochondrial oxidative phosphorylation deficiency in tumour cells; however, the enhanced glycolysis is insufficient to support cell proliferation without oxidative phosphorylation^2,3^. In fact, several studies have demonstrated that proliferating tumour cells generate a significant part of their energy through glutamine- and glucose-driven oxidative phosphorylation under normoxic as well as hypoxic conditions^4,5^. The highly tumourigenic and metastatic surviving cancer cells that are responsible for tumour relapse and are often referred to as cancer stem cells appear to be particularly reliant on oxidative phosphorylation for their energy provision^6–8^. Cancer cells devoid of mitochondrial DNA (mtDNA) are much more sensitive to cytotoxic drugs and unable to initiate tumours in animal models^9,10^. Remarkably, this loss of tumourigenicity can be restored by horizontal transfer of mtDNA from the host to the tumour cell^11^. Because mtDNA only codes for subunits of the oxidative phosphorylation enzymes and the RNA components required for their translation^12^, these observations demonstrate that oxidative phosphorylation is critical for tumour progression. It is, therefore, not surprising that oxidative phosphorylation is increasingly recognised as a potential target in cancer therapy^3,13^.

Already in the 1980s, we investigated the potential anti-tumour properties of tetracyclines based on the notion that these antibiotics inhibit mitochondrial protein synthesis. Tetracyclines block bacterial protein synthesis by binding to the small ribosomal subunit, thus preventing the attachment of aminoacyl-tRNAs^14^. Since mitochondrial ribosomes are evolutionarily related to bacterial ribosomes, tetracyclines also interfere with mitochondrial protein synthesis^15,16^. We showed that tetracyclines at concentrations used for anti-bacterial treatment cause a G_1_ cell cycle arrest *in vitro* and suppress the growth of various tumours in rodent models^17–19^. In addition, the tetracycline-derivative doxycycline delays tumour relapse after adriamycin or 1-β-d-arabinofuranosyl cytosine treatment of rat T-cell leukaemia and, under certain conditions, may result in a complete leukaemia eradication^20,21^. These findings were later supported by others who confirmed the proliferation arrest in G_1_ and inhibition of tumour growth in mouse xenograft models^22–26^. Furthermore, it has been shown that doxycycline decreases tumour-sphere formation efficiency of cancer stem cells^27–29^. We reported that patients with tumours of the nasopharynx and larynx who were treated with tetracyclines to prevent secondary bacterial infections survived longer than patients treated with erythromycin^30^. In a more recent clinical pilot study of breast cancer patients, pre-operative treatment with doxycycline decreased the expression of the stemness markers CD44 and ALDH1 in tumour biopsies, consistent with the view that doxycycline eliminates cancer stem cells *in vivo*^31^.

In the current *in vitro* study, we further examined the impact of doxycycline on cellular physiology to explain the *in vivo* observations. We compared the effects of doxycycline on the A549 human lung adenocarcinoma cell line and primary human dermal fibroblast. To confirm that the effects were indeed caused by inhibition of mitochondrial protein synthesis, we used the mtDNA-lacking A549 ρ° cell line as negative control. Key experiments were repeated in the COLO357 human pancreatic adenocarcinoma cell line and the HT29 human colon adenocarcinoma cell line. To investigate whether doxycycline treatment sensitises cancer cells to conventional anti-cancer agents, cells pretreated with doxycycline were exposed to the deoxycytidine analogue gemcitabine. Our experiments demonstrate that doxycycline-induced inhibition of mitochondrial protein synthesis decreases mitochondrial ATP generation, resulting in a slower proliferation rate of A549, COLO357 and HT29 cells. In addition, doxycycline treatment decreases the inner mitochondrial membrane potential (ΔΨ_m_) and produces oxidative stress, which together are likely to lower the apoptotic threshold for gemcitabine.

## Results

### Experimental approach

Fibroblast, A549, A549 ρ°, COLO357 and HT29 cells were seeded at an empirically determined density that allowed logarithmic growth over a 6-day period, without restriction by contact inhibition. One day after seeding, cultures were treated with vehicle or doxycycline for 5 days. In some experiments, gemcitabine was added during the last 2 days (Supplementary Fig. S1). Doxycycline was used at a concentration of 10 μg/ml, which corresponds to the serum level in patients receiving anti-bacterial medication with the standard recommended dose^32^. Gemcitabine was used at 10 ng/ml because a dose-response experiment indicated that this concentration decreased the total A549 cell number by half over a 2-day period (Supplementary Fig. S2).

### Mitochondrial protein synthesis and mtDNA copy number

**Figure 1:**
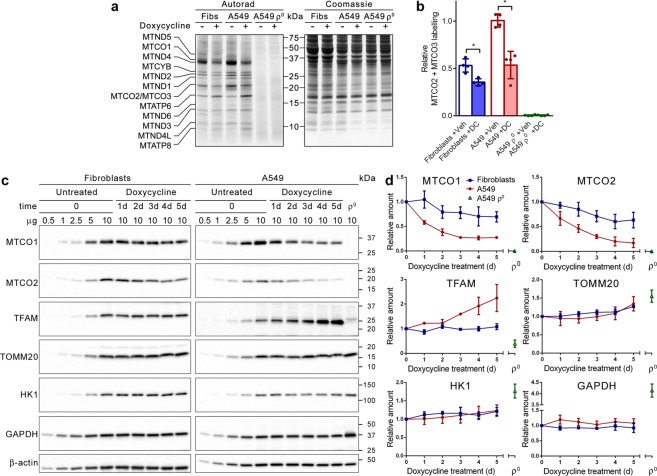
Doxycycline inhibits mitochondrial translation. (**a**) *De novo* mitochondrial protein synthesis of fibroblasts (Fibs), A549 and A549 ρ° cells treated with vehicle (−) or doxycycline (+). After 5 days of treatment, cultures were pulse-labelled with [^35^S]-methionine in the presence of emetine. Protein samples were subjected to gel electrophoresis. The gel was stained with Coomassie brilliant blue followed by autoradiography to reveal the labelled mitochondrial translation products, indicated on the left. The migration of protein standards is indicated in the centre. (**b**) Mean MTCO2 + MTCO3 labelling signals in vehicle (Veh) and doxycycline (DC)-treated cells relative to the mean value of vehicle-treated A549 cells (n = 4). (**c**) Western blot images of samples from fibroblasts and A549 cells treated with doxycycline over a 5-day period and from untreated A549 ρ° cells. To facilitate quantification, serial dilutions of untreated cells, harvested at t = 0, were also applied. Blots were probed with antibodies against the indicated proteins. Migration of protein standards is indicated on the right. Full-size blots are presented in Supplementary Fig. S11. (**d**) Mean amounts of the indicated proteins in the treated cells over a 5-day period and of untreated A549 ρ° cells, relative to the amount in the cells at t = 0 (n = 3). Error bars indicate standard deviations. Asterisks denote statistically significant differences (p < 0.05).

First, we investigated the effect of doxycycline on mitochondrial protein synthesis. Fibroblast, A549 and A549 ρ° cells were treated for 5 days with doxycycline or vehicle, followed by a 1-hour labelling with [^35^S]-methionine in the presence of doxycycline or vehicle and emetine to block cytosolic protein synthesis. Autoradiography of samples separated by gel electrophoresis showed labelling of the 13 mtDNA-encoded polypeptides in fibroblasts and A549 cells, but not in A549 ρ° cells (Fig. 1a). Doxycycline resulted in inhibition of *de novo* synthesis of most but not all mtDNA-encoded polypeptides. Quantification of the signals of the co-migrating cytochrome-*c* oxidase subunits MTCO2 and MTCO3 from four independent experiments indicated that the synthesis of these polypeptides decreased by ~30% in doxycycline-treated fibroblasts and by ~50% in doxycycline-treated A549 cells, compared to vehicle-treated cells (Fig. 1b). In contrast to the diminished synthesis of most mtDNA-encoded polypeptides, the synthesis of the ATP synthase subunits MTATP6 and MTATP8 increased markedly in doxycycline-treated cells (Fig. 1a).

It has been suggested that doxycycline affects the mtDNA copy number^33^. To verify this, fibroblast, A549 and A549 ρ° cells were treated for 5 days with doxycycline or vehicle, followed by DNA extraction and cellular mtDNA copy number measurement using real-time quantitative PCR. Our experiments demonstrated that doxycycline does not alter the mtDNA copy number (Supplementary Fig. S3).

### Mitochondrial and glycolytic enzyme protein levels

To determine the impact of doxycycline on the levels of the mtDNA-encoded cytochrome-*c* oxidase subunits MTCO1 and MTCO2, fibroblast, A549, COLO357 and HT29 cells were treated with the antibiotic for up to 5 days. Every day, samples were taken for analysis by western blot. Samples of A549 ρ° cells were loaded on the gels as negative control. To enable quantification of the signals, a dilution series of untreated samples taken at t = 0 were also loaded on the gels. The blots showed that the MTCO1 and MTCO2 levels progressively decreased during the 5-day treatment (Fig. 1c and Supplementary Fig. 4a). Quantification of three independent experiments revealed that the levels were decreased by 30–37% in fibroblasts, by 72–83% in A549 cells, by 80–84% in COLO357 cells and by 63–70% in HT29 cells after 5 days of treatment (Fig. 1d and Supplementary Fig. 4b,c).

Further analyses indicated that doxycycline treatment of fibroblasts had no effect on the amount of the mtDNA transcription factor TFAM; however, the amount of TFAM more than doubled in A549 cells after 5 days of treatment (Fig. 1c,d). TFAM was barely detectable in A549 ρ° cells and migrated slightly slower. Possibly, this band represents premature TFAM containing its presequence for mitochondrial delivery, while mature TFAM is degraded because it is not stabilised by its binding to mtDNA^34^. The levels of the translocase of outer mitochondrial membrane subunit 20 (TOMM20) seemed to increase slightly in fibroblasts (~25%) and A549 cells (~30%) towards the end of the treatment, whereas the TOMM20 level was elevated by 50% in A549 ρ° cells compared to untreated A549 cells (Fig. 1c,d). Lastly, we looked at two key enzymes of glycolysis to investigate if their levels changed in response to the decreased mitochondrial protein synthesis. Hexokinase 1 (HK1), which is the most ubiquitously expressed isoform of the four hexokinases and localises to the outer mitochondrial membrane, showed possibly a slight increase of ~20% in fibroblasts and A549 cells after 5 days of treatment, while glyceraldehyde 3-phosphate dehydrogenase (GAPDH) remained stable throughout the treatment (Fig. 1c,d). In A549 ρ° cells, the HK1 level was ~80% higher and the GAPDH level was ~300% higher than in untreated A549 cells.

### Cytochrome-*c* oxidase activity, citrate synthase activity and mitochondrial mass

**Figure 2:**
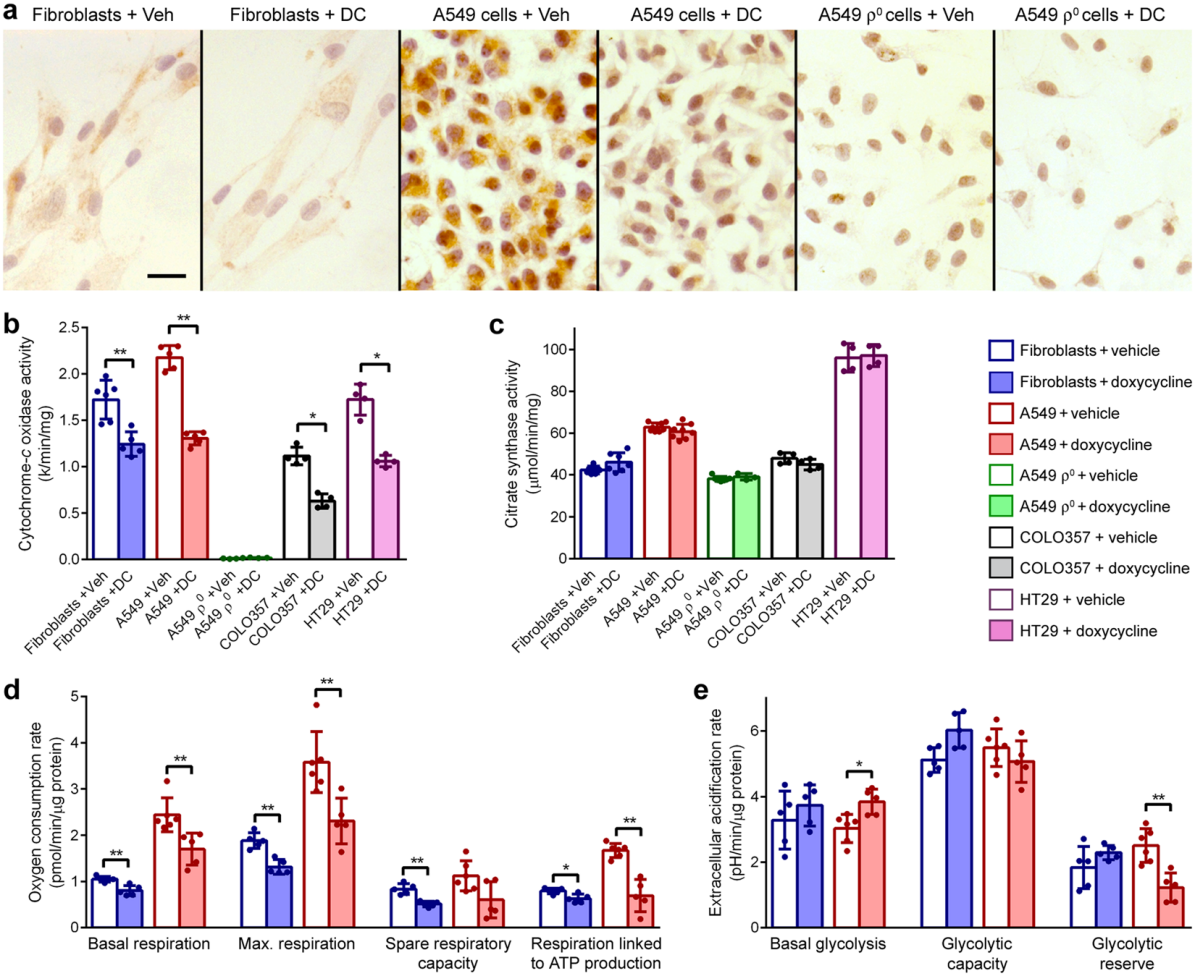
Doxycycline decreases oxidative phosphorylation. (**a**) Brown histochemical staining for cytochrome-*c* oxidase activity in fibroblasts, A549 and A549 ρ° cells treated with vehicle or doxycycline for 5 days. Cell nuclei were counterstained purple with haematoxylin. Scale bar represents 10 μm (**b,c**) Mean cytochrome-*c* oxidase and citrate synthase activity in lysates of cells treated with vehicle (Veh) or doxycycline (DC) for 5 days (n ≥ 4). (**d**) Mean basal respiration, maximal respiration, spare respiratory capacity and respiration coupled to ATP production in cells treated with vehicle or doxycycline for 5 days (n ≥ 5). (**e**) Mean basal glycolysis, glycolytic capacity and glycolytic reserve in cells treated with vehicle or doxycycline for 5 days (n ≥ 5). Error bars indicate standard deviations. Asterisks denote statistically significant differences (*p < 0.05; **p < 0.01).

To investigate if the drop in cytochrome-*c* oxidase subunit levels had an effect on the enzyme activity, fibroblast, A549 and A549 ρ° cells were treated for 5 days with doxycycline or vehicle, followed by brown histochemical staining for cytochrome-*c* oxidase activity. As expected, A549 ρ° cells showed no brown staining. Doxycycline-treated fibroblasts showed less brown staining than vehicle-treated fibroblasts but the difference was only just discernible (Fig. 2a). In contrast, all doxycycline-treated A549 cells showed clearly much less brown staining than vehicle-treated A549 cells, indicating that doxycycline lowers cytochrome-*c* oxidase activity more in A549 cells than in fibroblasts.

To quantify the cytochrome-*c* oxidase activity and extend the experiments to COLO357 and HT29 cells, we carried out spectrophotometric assays with lysates from cultures treated for 5 days with doxycycline or vehicle. These experiments revealed that doxycycline decreased cytochrome-*c* oxidase activity on average by 19% in fibroblasts, by 40% in A549 cells, by 44% in COLO357 cells and by 39% in HT29 cells compared to vehicle treatment (Fig. 2b). Conversely, doxycycline had no effect on the activity of the Krebs cycle enzyme citrate synthase (Fig. 2c). A549 ρ° cells showed no cytochrome-*c* oxidase activity but did show citrate synthase activity albeit lower than in A549 cells.

To further investigate if the decrease in cytochrome-*c* oxidase activity in doxycycline-treated cultures is matched by a decrease in mitochondrial mass, mitochondria in fibroblast, A549 and A549 ρ° cells were labelled with MitoTracker Green. Image analysis revealed no difference in the average mitochondrial mass per cell between vehicle-treated and 5-day doxycycline-treated cultures (Supplementary Fig. 5).

### Cellular respiration and glycolysis

To study the effect of doxycycline on cellular respiration and glycolysis, we performed extracellular flux assays on fibroblast, A549 and A549 ρ° cells with a Seahorse Analyzer. These assays determine oxygen consumption rates as measure of cellular respiration and extracellular acidification rates as measure of glycolytic flux. Representative respiration and glycolysis test profiles of cells treated for 5 days with doxycycline or vehicle are shown in Supplementary Fig. S6. As anticipated, A549 ρ° cells showed no oxygen consumption in respiration tests and did not respond to oligomycin A in glycolysis tests. The assays were repeated independently 5–6 times. The compiled data are summarised in Fig. 2d,e. The respiration tests demonstrated that basal respiration, maximal respiration and respiration coupled to ATP production were all lower in doxycycline-treated cells than in vehicle-treated cells. This decrease was more pronounced in A549 cells than in fibroblasts. For instance, respiration coupled to ATP production in doxycycline-treated A549 cells was ~60% lower than in vehicle-treated cells, while this was only ~20% lower in doxycycline-treated fibroblasts. The glycolysis tests showed no changes in basal glycolysis, glycolytic capacity and glycolytic reserve of fibroblasts. In A549 cells, basal glycolysis increased ~25% and glycolytic reserve decreased ~50% as result of doxycycline treatment but glycolytic capacity was unaffected.

### Cell growth and apoptosis

To study the effect of doxycycline on the proliferative rate, we determined the doubling time of fibroblast, A549 and A549 ρ° cultures over a 5-day treatment period with doxycycline or vehicle. Doxycycline increased the doubling time of fibroblasts by 5% and that of A549 cells by 11% but the doubling time of A549 ρ° cells did not change (Supplementary Fig. S7a). We also measured the fraction of cells that were passing through S-phase after a 5-day treatment by labelling of replicating DNA with 5-bromo-2′-deoxyuridine (BrdU; Supplementary Fig. S8). The compilation of four independent experiments demonstrated that doxycycline had no effect on fibroblasts or A549 ρ° cells but caused a 43% decrease of A549 cells passing through S-phase (Supplementary Fig. S7b).

**Figure 3:**
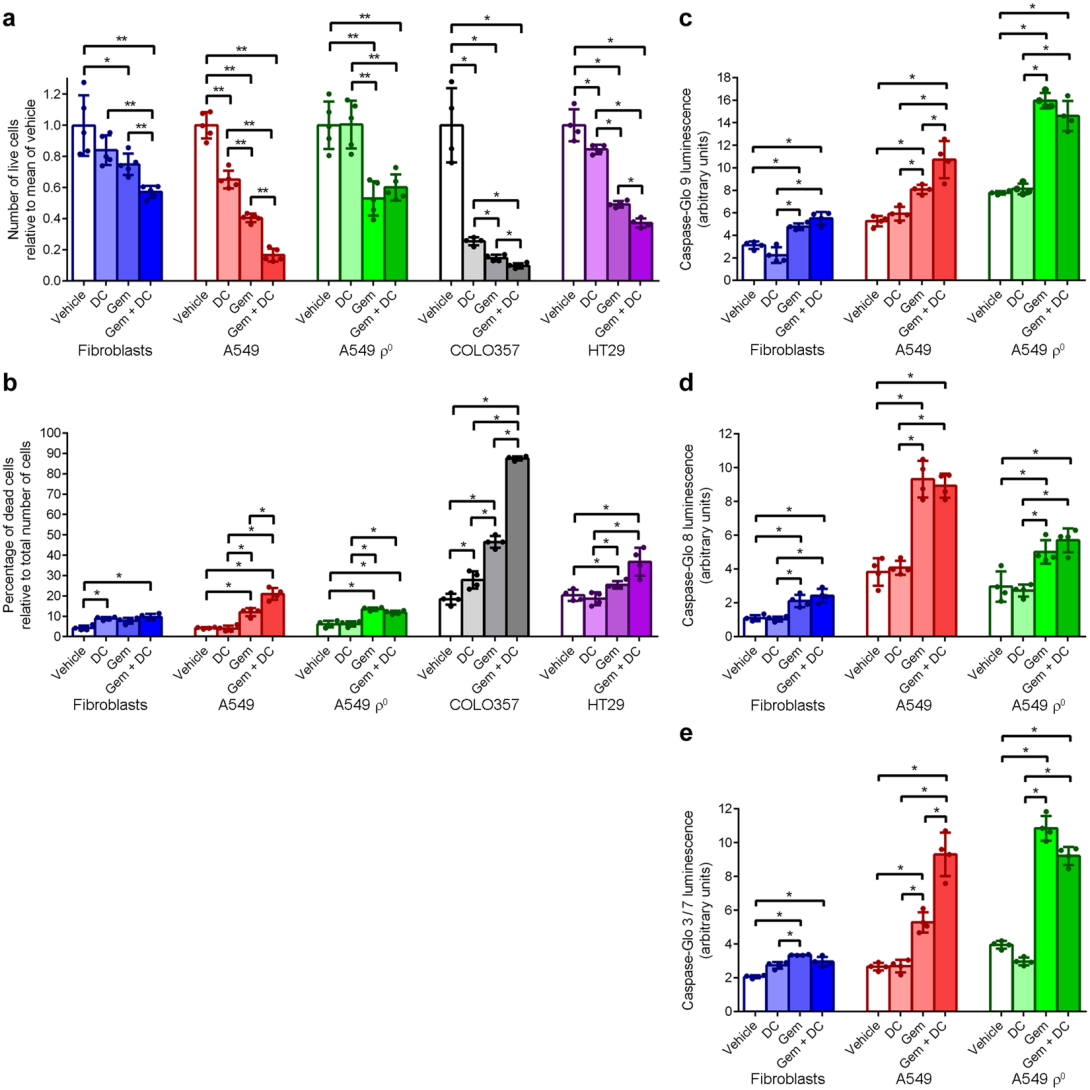
Doxycycline increases the inhibitory effect of gemcitabine on cell growth and the inducing effect of gemcitabine on caspase 9 and 3/7 activities. Fibroblast, A549, A549 ρ^0^, COLO357 and HT29 cells were treated with vehicle or doxycycline (DC) for 5 days. During the last 2 days, cultures were not co-treated or co-treated with gemcitabine (Gem). (**a**) Mean number of live cells after 5 days of treatment, relative to vehicle-treated cells (n ≥ 4). (**b**) Percentage of dead cells relative to total cell number after 5 days of treatment (n = 4). (**c**) Mean caspase 9 activity after 5 days of treatment, as determined in Caspase-Glo 9 luminescence activity assays (n = 4). (**d**) Mean caspase 8 activity after 5 days of treatment, as determined in Caspase-Glo 8 luminescence activity assays (n = 4). (**e**) Mean caspase 3/7 activity after 5 days of treatment, as determined in Caspase-Glo 3/7 luminescence activity assays (n = 4). Error bars indicate standard deviations. Asterisks denote statistically significant differences (*p < 0.05; **p < 0.01).

To investigate if co-treatment with doxycycline enhances the cytotoxic effect of gemcitabine, cells were treated with vehicle for 5 days, or doxycycline for 5 days, or vehicle for 3 days followed by gemcitabine for 2 days, or doxycycline for 3 days followed by doxycycline and gemcitabine co-treatment for 2 days (Supplementary Fig. S1). Cell counting of fibroblast cultures after the treatments indicated that doxycycline had no significant effect on the total cell number, while gemcitabine decreased the cell number on average by 25% (Fig. 3a). Co-treatment of fibroblasts with doxycycline and gemcitabine resulted in a further decrease of surviving fibroblasts by 24% compared to cells treated with gemcitabine alone. Doxycycline decreased the A549 cell number by 35%, whereas gemcitabine decreased the A549 cell number by 60%. Co-treatment of A549 cells with doxycycline and gemcitabine resulted in a further decrease of surviving A549 cells by 59% compared to cells treated with gemcitabine alone. Doxycycline had no effect on A549 ρ° cell numbers but gemcitabine decreased A549 ρ° cell numbers by 47%. COLO357 and HT29 cells showed a similar pattern of cell number decreases in response to the treatments as A549 cells (Fig. 3a). The 74% decrease in mean COLO357 cell number as result of 5-day doxycycline treatment was particularly striking.

To examine the effects of the treatments on cell death, we determined the fraction of dead cells relative to the total number of cells after the four treatments (Fig. 3b). Doxycycline or doxycycline with gemcitabine treatment of fibroblasts resulted in a small (4–5%) increase in the fraction of dead cells, compared to untreated cells. In the cancer cell lines, doxycycline treatment had no effect on cell death, except for COLO357 cells where the antibiotic resulted in an increase of the fraction of dead cells by 9% compared to vehicle-treated cells. Gemcitabine treatment caused a significant 5–28% increase in the fraction of dead cells in the cancer cell lines compared to vehicle-treated cultures. This increase was further boosted by co-treatment with doxycycline in A549 and COLO357 cultures, but co-treatment had no significant additional effect on A549 ρ° and HT29 cultures.

To explain the decreased cell survival observed during co-treatment with doxycycline and gemcitabine, we reasoned that doxycycline may lower the apoptotic threshold. We investigated this by measuring caspase 9, 8 and 3/7 activities in fibroblast, A549, and A549 ρ° cultures after the 5-day treatment protocol. The initiator caspase 9 plays a key role in the intrinsic apoptotic pathway, which is triggered by mitochondrial stress. The initiator caspase 8 has a critical function in the extrinsic apoptotic pathway, which is set off by extracellular ligands via cell surface death receptors. Activated initiator caspases activate the downstream executioner caspases 3 and 7, which degrade cellular components to induce apoptosis^35^. Compared to vehicle treatment, doxycycline treatment did not affect caspase 9, 8 or 3/7 activities in any of the cell types but gemcitabine treatment caused an increase of caspase 9, 8 and 3/7 activities in all three cell types (Fig. 3c–e). The gemcitabine-induced increase in activities was quite modest in fibroblasts, more pronounced in A549 cells and for caspase 9 and 3/7 even more prominent in A549 ρ° cells. Compared to gemcitabine treatment alone, doxycycline and gemcitabine co-treatment did not result in a further increase in caspase 9, 8 and 3/7 activities in fibroblasts and A549 ρ° cells. Caspase 8 activity showed also no additional increase in co-treated A549 cells but caspase 9 and 3/7 activities increased further compared to A549 cells treated with gemcitabine alone. These results support the view that doxycycline treatment does not induce apoptosis but lowers the threshold for the intrinsic apoptotic pathway in A549 cells, since doxycycline treatment alone does not increase the caspase activities, whereas doxycycline and gemcitabine co-treatment results in markedly higher caspase 9 and 3/7 activities than gemcitabine treatment alone. The extrinsic apoptotic pathway is not affected by doxycycline because doxycycline and gemcitabine co-treatment causes no additional increase in caspase 8 activity compared to gemcitabine treatment alone.

### ATP levels

**Figure 4:**
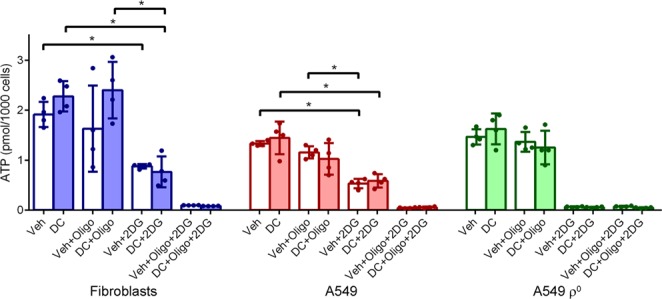
Doxycycline has no effect on cellular ATP levels synthesized through glycolysis or oxidative phosphorylation. Fibroblast, A549 and A549 ρ^0^ cultures were treated with vehicle (Veh) or doxycycline (DC) for 5 days. To determine the contribution of glycolysis and oxidative phosphorylation to the cellular ATP pool, cultures were subsequently left untreated or were treated with the oxidative phosphorylation inhibitor oligomycin A (Oligo) and/or the glycolysis inhibitor 2-deoxyglucose (2DG) directly prior to the ATP assays. Bars represent the mean cellular ATP levels (n = 4). Error bars indicate standard deviations. Asterisks denote statistically significant differences (p < 0.05).

The threshold of the intrinsic apoptotic pathway may be lowered by a decrease of ATP levels or ΔΨ_m_, or an increase of oxidative stress^36^. First, we investigated if ATP levels were affected by doxycycline. Total cellular ATP levels were determined in fibroblast, A549, and A549 ρ° cultures treated for 5 days with doxycycline or vehicle. Shortly before the measurements, cells were additionally treated with vehicle, the ATP synthase inhibitor oligomycin A, the hexokinase inhibitor 2-deoxy-d-glucose or both to assess the contribution of glycolysis and oxidative phosphorylation to the maintenance of ATP levels. We found no difference in ATP levels in doxycycline-treated cells compared to vehicle-treated cells for any of the three cell types (Fig. 4). Addition of oligomycin A had no effect on ATP levels, implying that glycolysis is able to offset the loss of ATP production by oxidative phosphorylation under the high glucose culture conditions. On the other hand, addition of 2-deoxyglucose resulted in a fall of ATP levels, indicating that oxidative phosphorylation is not able to fully compensate the loss of glycolytic ATP production through oxidation of glutamine from the medium via the Krebs cycle^37^. Unsurprisingly, addition of 2-deoxyglucose to A549 ρ° cells, or addition of both inhibitors to any of the cell types caused a complete loss of cellular ATP.

### Inner mitochondrial membrane potential (ΔΨ_m_)

**Figure 5:**
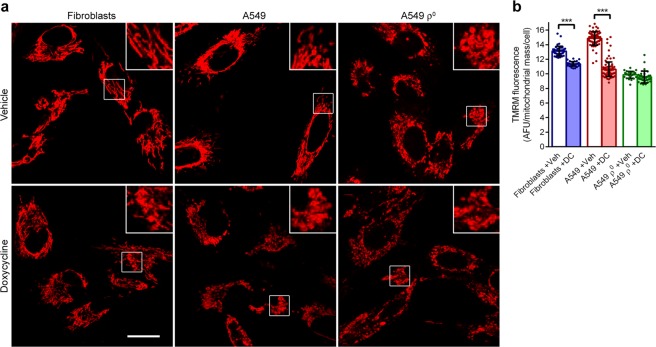
Doxycycline causes mitochondrial fragmentation and decreases ΔΨ_m_. Fibroblast, A549 and A549 ρ^0^ cultures were treated with vehicle (Veh) or doxycycline (DC) for 5 days, followed by staining with the ΔΨ_m_-dependent dye TMRM. (**a**) Representative fluorescent micrographs of TMRM-stained cells. Scale bar: 10 μm. Inserts show mitochondrial morphology. (**b**) Mean TMRM fluorescence per mitochondrial mass per cell (n ≥ 36) in arbitrary fluorescent units (AFU). Error bars indicate standard deviations. Asterisks denote statistically significant differences (p < 0.005).

Next, we investigated whether ΔΨ_m_ is affected by doxycycline with tetramethylrhodamine methyl ester (TMRM). This red fluorescent dye accumulates in mitochondria proportional to ΔΨ_m_. Microscopic examination of fibroblast, A549, and A549 ρ° cultures treated for 5 days with doxycycline or vehicle and loaded with TMRM revealed that doxycycline resulted in swelling and fragmentation of the mitochondrial tubular reticulum in fibroblasts and A549 cells (Fig. 5a). Mitochondria of vehicle-treated A549 ρ° cells had a swollen appearance similar to doxycycline-treated A549 cells. Treatment of A549 ρ° cells with doxycycline had no further effect on mitochondrial morphology. Quantification of the mitochondrial fluorescence per cell revealed that the mean staining intensity was 14% lower in doxycycline-treated fibroblasts than in vehicle-treated cells, whereas this was 28% lower in doxycycline-treated A549 cells (Fig. 5b). It indicates that ΔΨ_m_ is more affected by doxycycline in A549 cells than in fibroblasts, while the antibiotic had no influence on ΔΨ_m_ in A549 ρ° cells.

### Oxidative stress

**Figure 6:**
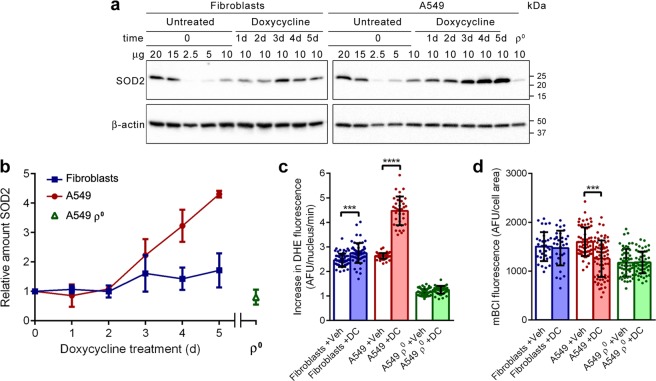
Doxycycline causes oxidative stress in A549 cells. (**a**) Western blot images of samples from fibroblasts and A549 cells treated with doxycycline over a 5-day time period and untreated A549 ρ^0^ cells. To facilitate quantification, serial dilutions of untreated cells, harvested at t = 0, were also applied. Blots were probed with antibodies against SOD2 and β-actin. Migration of protein standards is indicated on the right. Full-size blots are presented in Supplementary Fig. S12. (**b**) Mean amounts of SOD2 in the treated cells over a 5-day time period and untreated A549 ρ^0^ cells, relative to the amount in cells at t = 0 (n = 3). (**c**) Fibroblast, A549 and A549 ρ^0^ cultures were treated with vehicle (Veh) or doxycycline (DC) for 5 days, followed by staining with the ROS indicator dye DHE. Bars represent the mean DHE fluorescence increase in arbitrary fluorescent units (AFU) in the nucleus per minute (n ≥ 42). (**d**) Fibroblast, A549 and A549 ρ^0^ cultures were treated with vehicle or doxycycline for 5 days, followed by staining with the GSH indicator dye mBCI. Bars represent the mean mBCI fluorescence in the cell (n ≥ 33). Error bars indicate standard deviations. Asterisks denote statistically significant differences (***p < 0.005, ****p < 0.001).

In the final experiments, we looked at three aspects of oxidative stress. Firstly, we determined the levels of mitochondrial superoxide dismutase SOD2 in fibroblast, A549, and A549 ρ° cultures treated for up to 5 days with doxycycline. Western blots suggested that SOD2 levels increased somewhat in fibroblasts during the last 3 days of treatment (Fig. 6a,b). In doxycycline-treated A549 cells, the increase of SOD2 levels was unquestionable; it more than quadrupled over the last 3 days of treatment, indicating that SOD2 expression is induced by doxycycline in A549 cells after a lag phase of 2 days. In A549 ρ° cells, SOD2 levels were markedly lower than in untreated A549 cells.

Subsequently, we examined with dihydroethidium (DHE) if doxycycline affects the rate of reactive oxygen species (ROS) production in the cytosol. Upon oxidation by a superoxide anion, DHE is converted into 2-hydroxyethidium, while upon oxidation by a hydroxyl radical or peroxynitrite, DHE is converted to ethidium^38^. Both reaction products relocate from the cytosol to the nucleus, intercalate into DNA and exhibit red fluorescence. Microscopic inspection of cultures treated for 5 days with doxycycline or vehicle and loaded with DHE showed red fluorescent-stained nuclei (Supplementary Fig. S9a). Measurement of the increase in fluorescent staining intensity per cell per minute indicated that the mean rate increase was 12% higher in doxycycline-treated fibroblasts than in vehicle-treated cells, while this mean rate increase was 69% higher in doxycycline-treated A549 cells (Fig. 6c). It implies that doxycycline increased the ROS production much more in A549 cells than in fibroblasts. Doxycycline had no impact on the ROS production of A549 ρ° cells.

Lastly, we assessed the levels of glutathione (GSH), the main cellular antioxidant, with the blue fluorescent probe monochloro-bimane (mBCI)^39^. Microscopic examination of cell cultures treated for 5 days with doxycycline or vehicle and loaded with mBCI revealed blue fluorescent cells (Supplementary Fig. S9b). Quantification of the fluorescent staining intensity showed that the mean cellular intensity was 22% lower in doxycycline-treated A549 cells than in vehicle-treated cells, whereas the staining intensity was not affected by doxycycline in fibroblasts or A549 ρ° cells (Fig. 6d). Thus, it appears that doxycycline lowers the GSH levels in A549 cells but not in fibroblasts or A549 ρ° cells.

## Discussion

The chemotherapeutic drug gemcitabine is widely used to treat a large number of cancer types. Although it is one of the most effective medications for first-line treatment, as a salvage regimen for patients with relapsed or refractory cancer its success is limited^40^. Resistance of pancreatic tumours to gemcitabine has been shown to be dependent on mitochondria-mediated apoptosis^41^. Cancer cell mitochondria have an unusually high intrinsic ΔΨ_m_^42^. ΔΨ_m_ correlates strongly with cancer malignancy^43,44^. The high ΔΨ_m_ may facilitate evasion of the cytotoxic effects of gemcitabine because an increase in ΔΨ_m_ is likely to raise the apoptotic threshold of a cell. Therefore, targeting of mitochondria may provide an effective means to circumvent the resistance of cancer cells to apoptosis. Tetracyclines have been used for > 50 years to treat or prevent bacterial infections. We have shown that tetracyclines suppress tumour growth and delay tumour relapse in rodent xenograft models^18–21^. The rationale of the use of tetracyclines was that these antibiotics inhibit mitochondrial protein synthesis, leading to a decrease of mtDNA-encoded oxidative phosphorylation subunit levels and, as a consequence, a decrease of mitochondrial ATP production, thus inhibiting cell proliferation, which is an energy demanding process. In the present study, we investigated the effects of doxycycline on cancer cell line and primary fibroblast cultures to further dissect the mechanisms by which this tetracycline derivative exerts its anti-cancer effect. We confirmed that doxycycline inhibits mitochondrial protein synthesis and demonstrated that this causes a decrease of the mitochondrial energy generating capacity, especially in cancer cells. This results in a markedly slower proliferation rate of cancer cells but not of fibroblasts. Furthermore, we found that doxycycline treatment lowers the ΔΨ_m_ and causes oxidative stress, which collectively are likely to lower the apoptotic threshold for gemcitabine and result in increased cell death in gemcitabine-treated cells (Supplementary Fig. S10).

In the experiments, cancer cells were always considerably more affected by doxycycline than fibroblasts. We detected lower levels of the mitochondrially synthesised oxidative phosphorylation subunits in doxycycline-treated cancer cells, which we assume is the result of the higher proliferation rate of the cancer cells that will cause a faster depletion of these proteins through cell division. The ATP production coupled to mitochondrial respiration showed a larger decrease in doxycycline-treated A549 cells, which will be a direct result of the lower levels of mitochondrially synthesised proteins. The ΔΨ_m_ of A549 cells dropped more during doxycycline treatment than in fibroblasts. The most likely explanation for this is that the oxidative phosphorylation enzymes that maintain ΔΨ_m_ deplete faster in A549 cells. The more severe depletion of oxidative phosphorylation enzymes, and the fact that superoxide production depends critically on the NADH/NAD^+^ ratio in the mitochondrial matrix^45^, probably explain the strong increase in oxidative stress in doxycycline-treated A549 cells since NADH/NAD^+^ will be higher as the mitochondria will use less NADH to drive oxidative phosphorylation. The apparent selectivity of doxycycline for cancer cells is supported by its limited side effects in patients. Phototoxicity, hyperpigmentation and photo-onycholysis have been reported to be associated with doxycycline use^46–48^ but, overall, doxycycline is well-tolerated and there is no reason to exclude doxycycline as potential anti-cancer drug.

Some authors have attributed the anti-tumour effect of doxycycline on its inhibitory action of matrix metalloproteases^22,23,49,50^. We used the mtDNA-lacking A549 ρ° cell line as negative control. All effects of doxycycline observed in the parental A549 cell line and primary fibroblasts were not seen in A549 ρ° cells. This proves that the effects were caused by inhibition of mitochondrial protein synthesis.

To assess the effect of doxycycline on mitochondrial protein synthesis, we labelled the mitochondrial translation products with [^35^S]-methionine, followed by separation on gels and autoradiography. Others have used radioactive assays followed by scintillation counting to measure doxycycline-mediated inhibition of mitochondrial protein synthesis^15,51^ but, to our knowledge, autoradiographs with samples from doxycycline-treated cells showing the individual mitochondrial translation products have not been documented earlier, except in our recent publication^52^. We found that the synthesis of 11 of the 13 mitochondrial translation products was inhibited by doxycycline, while the synthesis of two mitochondrial translation products, MTATP6 and MTATP8, was increased. We do not know the mechanism producing this increase but we suspect that it involves a response to the long-term exposure of doxycycline because in earlier experiments, in which the cells were exposed to doxycycline for 2 hours, this increase was not noted^52^. The inhibition of the synthesis of the 11 mitochondrial translation products was modest but dose-response experiments by others have indicated that the 10 μg/ml of doxycycline we used results in maximum inhibition^51^.

Western blot analyses revealed a progressive loss of the mitochondrially encoded proteins MTCO1 and MTCO2 during the 5-day doxycycline treatment. Others have reported comparable results^16^. In addition, we found that TFAM levels more than doubled in A549 cells during the treatment, whereas TFAM remained stable in fibroblasts. This may be a compensatory mechanism of the cancer cells in an attempt to boost mitochondrial protein expression. Levels of TOMM20 remained relatively stable over the treatment period in both cell types, suggesting that the mitochondrial mass was not affected by doxycycline. This notion is supported by the matching mitochondrial MitoTracker Green staining and citrate synthase activities in treated and untreated cells. We also investigated the levels HK1 and GAPDH on western blots. Both proteins remained relatively stable during the treatment, suggesting that these glycolytic enzymes are not substantially upregulated in response to the decreased oxidative phosphorylation. In the extracellular flux assays, the basal glycolysis was slightly higher and the glycolytic reserve was lower in doxycycline-treated A549 cells than in untreated cells; however, the total glycolytic capacity was unaffected, corroborating the western blot results. The extracellular flux assays further demonstrated that doxycycline treatment decreases mitochondrial basal respiration, maximal respiration and respiration coupled to ATP production. Impairment of basal respiration by tetracyclines has also been shown by others^16,53,54^.

The doubling time of A549 cells measured over the 5-day doxycycline treatment period showed a modest increase of 11% compared to vehicle-treated cells, whereas after 5 days of doxycycline treatment the number of A549 cells passing through S-phase was 43% down. This seeming contradiction can be explained by a lag phase before an effect of doxycycline on the proliferation rate becomes apparent because the levels of mitochondrially encoded oxidative phosphorylation subunits are initially still relatively high. The western blots showed that the doxycycline-induced decrease of MTCO1 and MTCO2 in A549 cells flattens after 3 days of treatment. Earlier, we showed that a proliferation slowdown of A549 cells occurs after 3 days of doxycycline treatment^52^. Apparently, at this point, the mitochondrial energy generating capacity is too low to maintain normal cell proliferation. For this reason, the time point to start gemcitabine co-treatment was chosen after 3 days of doxycycline treatment.

Several groups have reported that doxycycline induces apoptosis in other cancer cell lines at higher doxycycline concentrations than used here^24,49,50,55^. We found that doxycycline treatment did not induce caspase 9, 8 or 3/7 activity in A549 cells, supporting our earlier observations that doxycycline does not cause activation of procaspase 3^52^. However, doxycycline and gemcitabine co-treatment resulted in a strong induction of caspase 9 and 3/7 activity when compared to gemcitabine alone. Thus, it appears that doxycycline treatment primes the mitochondria to undergo apoptosis, opening a new therapeutic window. To investigate the basis for this priming, we assessed cellular ATP levels, ΔΨ_m_ and oxidative stress, which are known to control the threshold of the mitochondria-mediated apoptotic pathway^36^. We found that ATP levels were not affected by doxycycline. We expect that cells will try to maintain their ATP levels and adjust their metabolic rate according to the ATP synthesis rate. Consequently, the decreased oxidative phosphorylation causes a deceased proliferation rate. Assessment of ΔΨ_m_ with TMRM indicated that doxycycline decreases ΔΨ_m_. Despite the absence of mtDNA, A549 ρ° cells contained polarised mitochondria. This ΔΨ_m_ is thought to arise from the electrogenic exchange of ATP^4-^ for ADP^3-^ by the adenine nucleotide carrier^56^. The TMRM staining further revealed swollen, fragmented mitochondria in doxycycline-treated cells. This has also been seen by others^16^ and indicates a shift to mitochondrial fragmentation, which may serve as prelude to mitophagy.

Because of signalling and metabolic alterations, cancer cells are inherently under increased oxidative stress^57,58^. It has been shown that this characteristic makes cancer cells more vulnerable to free radical-induced apoptosis when challenged by further oxidative stress^57,59^. Our experiments revealed that doxycycline induces the expression of SOD2 in A549 cells. Despite the stimulated antioxidant defence, cells are apparently unable to counteract the elevated ROS production because we observed that ROS dramatically increases in doxycycline-treated A549 cells, while GSH decreases. We expect that the increased oxidative stress contributes to the predisposition of the cancer cells to apoptosis.

In addition to the priming of cancer cells to undergo apoptosis, doxycycline may have other benefits. Drug-resistant cancer cells often acquire their resistance by over-expression of ATP-driven efflux pumps that expel the drug before it inflicts damage. A doxycycline-induced oxidative phosphorylation deficiency will limit the ATP supply and may curtail pump function. Furthermore, it has been shown that intratumour bacteria in colon cancer models deactivate gemcitabine by deamination of the deoxycytidine moiety^60^. Combination therapy with doxycycline may offer a therapeutic advantage for tumours carrying intratumour bacteria, provided that these are doxycycline-sensitive.

## Methods

### Cell culturing and counting

A primary human dermal fibroblast culture was established from a skin explant of a 27-year-old female control subject according to standard procedures. The donor provided prior informed written consent. Ethical approval for this work was obtained from the Royal Free Hospital and Medical School Research Ethics Committee (REC 07/H0720/161) in compliance with national legislation and the Declaration of Helsinki. The A549 human non-small-cell alveolar adenocarcinoma cell line and the HT29 human colon epithelium adenocarcinoma cell line were purchased from the European Collection of Authenticated Cultures. The COLO357 human pancreatic adenocarcinoma cell line was a gift from the Royal Free Hospital Department of Surgery, London^61^. The mtDNA-less A549 ρ° cell line was generated by prolonged culturing in the presence of 50 ng/ml of ethidium bromide. Depletion of mtDNA was confirmed by PCR. Cells were cultured in 10-cm tissue culture dishes in Dulbecco’s modified Eagle’s medium (DMEM) containing GlutaMAX and 25 mM glucose (Gibco Lifetechnologies, 61965029), supplemented with 10% foetal bovine serum, 1 mM sodium pyruvate, 50 μg/ml of uridine, 50 units/ml of penicillin and 50 μg/ml of streptomycin at 37 °C in a humidified atmosphere of 5% CO_2_ in 95% air unless stated otherwise. Cultures were checked regularly for mycoplasma infection.

For counting, cells were dislodged by trypsinisation, collected by centrifugation and resuspended in culture medium followed by trypan blue exclusion cell counting on C-Chip haemocytometer slides (NanoEnTek) in duplicate. Cell growth counting experiments were carried out in 4–5 fold with independent samples. The doubling time (DT) of the cultures was calculated with the formula DT = (t-t_0_)log2/(logN-logN_0_), where t and t_0_ are the times when the cells were counted and N and N_0_ are the cell numbers at those times, respectively.

### Labelling of mitochondrial translation products

To reveal the effect of doxycycline on *de novo* mitochondrial protein synthesis, 7.0 × 10^4^ fibroblasts, 1.0 × 10^4^ A549 cells or 3.0 × 10^4^ A549 ρ° cells were seeded on 10-cm tissue culture dishes. From the day after seeding, cells were treated daily with 10 μg/ml (19.5 μM) of doxycycline or vehicle (water) over a 5-day period. Doxycycline was added to fresh culture medium from a 10 mg/ml stock of doxycycline hyclate (Sigma-Aldrich) in water, which was stored at −20 °C and protected from light. After 4 days of treatment, A549 and A549 ρ° cells were replated at ~50% confluency in wells of a 6-well tissue culture plate and treatment was continued. The next day, when ~85% confluency was reached, mitochondrial translation products were labelled with l-[^35^S]-methionine (Perkin Elmer), followed by protein extraction, resolution on polyacrylamide gels, Coomassie staining and autoradiography as described^52^. Images of the stained gels and autorads were captured with the BioRad Chemidoc MP Imaging System. Signals were quantified with BioRad Image Lab 5.1 software. Radioactive signals on the autorad were normalised with the help of the total Coomassie staining signal in the gel lanes and expressed relative to the radioactive signal from A549 cells treated with vehicle. Experiments were performed in quadruplicate with independent samples.

### Determination of mtDNA copy number

Logarithmically growing cell cultures were treated daily with 10 μg/ml of doxycycline or vehicle in fresh medium. After 5 days of treatment, cells were dislodged by trypsinisation and collected by centrifugation, followed by isolation of total DNA with the Puregene Corekit A (Qiagen). To determine the mtDNA copy number in the samples, two separate real-time quantitative PCR amplifications were carried out: the forward 5′-CATCTGGTTCCTACTTCAGGG and reverse 5′- TGAGTGGTTAATAGGGTGATAGA primers were used to amplify part of the mtDNA D-loop region, and the forward 5′-TGCTGTCTCCATGTTTGATGTATCT and reverse 5′-TCTCTGCTCCCCACCTCTAAGT primers were used to amplify part of the nuclear single copy β2-microglobulin gene (*B2M*)^62^. Amplifications were performed in quadruplicate on a Step One Real-Time PCR System (Thermo Fisher Scientific). Reactions of 20 μl contained 1× Power SYBR Green PCR master mix (Thermo Fisher Scientific), 0.3 μM forward primer, 0.3 μM reverse primer and 8 ng of DNA. The PCR program consisted of an enzyme activation step of 10 minutes at 95 °C, followed by 45 cycles of a denaturing step of 15 seconds at 95 °C, an annealing and elongation step of 1 minute at 60 °C, and a reading of the fluorescence. The mtDNA copy number was calculated with the formula: 2 × 2^−(Ct mtDNA - Ct nDNA)^, where Ct _mtDNA_ is the cycle threshold for mtDNA and Ct _nDNA_ is the cycle threshold for nuclear DNA.

### Western blot analysis

To examine the effect of doxycycline on the levels of mitochondrial and glycolytic enzyme proteins, 7.0 × 10^4^ fibroblasts, 1.0 × 10^4^ A549 or COLO357 or HT29 cells, or 3.0 × 10^4^ A549 ρ° cells were seeded on multiple 10-cm tissue culture dishes. From the day after seeding (t = 0), the cells were treated daily with 10 μg/ml of doxycycline in fresh culture medium over a 5-day period. Dishes were chosen randomly each day for harvesting of cells by trypsinisation. Cells collected by centrifugation were extracted with 1% Triton X-100 and 10 μg of extracted protein samples were analysed on western blots as described^52^, except that samples were resolved on Mini-Protean TGX 4‒20% gels (BioRad Laboratories). To facilitate quantification, serial dilutions of extracts from (untreated) cells at t = 0 were also loaded on the gels. The following antibodies were used for detection: anti-MTCO1 (Abcam, ab140705), anti-MTCO2 (Abcam, ab110258), anti-TFAM (Thermo Fisher Scientific, MA5-16148), anti-TOMM20 (Santa Cruz Biotechnology, sc-11415), anti-HK1 (Abcam, ab150423), anti-GAPDH (Abcam, ab8245), anti-SOD2 (Santa Cruz Biotechnology, sc-30080) and anti-β-actin (Abcam, ab6276). Signals were quantified with BioRad Image Lab 5.1 software. The relative amount of protein was calculated with the help of a calibration curve constructed from the dilutions series, corrected for uneven loading with the aid of the relative amount of β-actin, and expressed relative to the amount in cells at t = 0. Experiments were performed in triplicate with independent samples.

### Cytochrome-*c* oxidase and citrate synthase activity assays

To investigate the effect of doxycycline on cytochrome-*c* oxidase activity, cells were seeded at low density on poly-l-lysine-coated glass coverslips and cultured in 6-well plates. From the day after seeding, cells were treated daily with 10 μg/ml of doxycycline or vehicle in fresh medium. After 5 days of treatment, cells were stained histochemically for cytochrome-*c* oxidase activity^63^. Coverslips were washed four times with calcium and magnesium-supplemented phosphate-buffered saline (PBS; Thermo Fisher Scientific), drained and briefly air-dried. Then, coverslips were incubated in 50 mM sodium phosphate buffer (pH 7.4), 0.5 mg/ml of 3,3′-diaminobenzidine (Sigma-Aldrich), 1 mg/ml of equine heart cytochrome-*c* (Sigma-Aldrich) and 2 μg/ml of catalase (Sigma-Aldrich) for 1 hour at 37 °C. This was followed by two washes in PBS, a 10-minute treatment with Quick DAB Enhancer (Innovex Biosciences) and another two washes with PBS. Nuclei were counterstained for 2 minutes with Meyer’s haematoxylin solution (Sigma-Aldrich), washed in water, destained with 70% ethanol, 0.5% HCl and washed again in water. Coverslips were dehydrated in three dishes with ethanol, cleared in three dishes with xylene and mounted on microscope slides with DPX mountant (Sigma-Aldrich). Coverslips were examined under a Zeiss Axiophot microscope. Images were captured with a Zeiss Axiocam MRm camera and were processed with Adobe Photoshop software. Experiments were performed in quadruplicate.

In addition, cytochrome-*c* oxidase and citrate synthase activities were determined spectrophotometrically in lysates of logarithmically growing cell cultures treated daily with 10 μg/ml of doxycycline or vehicle in fresh medium over a 5-day period. After 5 days of treatment, cells were dislodged by trypsinisation, collected by centrifugation in culture medium, washed with homogenisation buffer (10 mM Tris∙HCl (pH 7.4), 320 mM sucrose, 1 mM EDTA, 1 mM phenylmethylsulfonyl, 1 μg/ml of leupeptin and 1 μg/ml of pepstatin A) at 4 °C and lysed in homogenisation buffer containing 1% *n*-dodecyl-β-d-maltoside (Anatrace) on ice. Assays were performed in 4–8-fold as described^52^.

### Extracellular flux analysis

Extracellular flux analysis was carried out on the Seahorse XFp platform (Agilent Technologies) as recommended by the supplier. Logarithmically growing cell cultures were treated daily with 10 μg/ml of doxycycline or vehicle in fresh medium. After 4 days of treatment, 1.5 × 10^4^ fibroblasts, or 1.2 × 10^4^ A549 or A549 ρ° cells were replated in wells of an XFp cell culture microplate and cultured with 10 μg/ml of doxycycline or vehicle in 80 μl of medium for one day. To analyse oxidative phosphorylation function, the medium in the wells was replaced with 175 μl XF Base medium (Agilent Technologies) pH 7.4 (NaOH), containing 10 mM d-(+)-glucose, 1 mM sodium pyruvate, 2 mM l-glutamine, with or without 10 μg/ml of doxycycline. After 1 hour of humidified incubation at 37 °C, oxidative phosphorylation was evaluated on the XFp platform with the Seahorse XFp Mito Stress Test Kit (Agilent Technologies). After three measurements under basal conditions, oligomycin A was injected to a final concentration of 1 μM followed by three measurements. Then, carbonyl cyanide 4-(trifluoromethoxy)phenylhydrazone (FCCP) was injected to a final concentration of 1 μM for A549 and A549 ρ° cells or 2 μM for fibroblasts, followed by three measurements. Last of all, rotenone and antimycin A were injected, both to a final concentration of 1 μM, followed by three measurements.

To analyse glycolytic function, the medium in the wells of the XFp cell culture microplate was replaced with 175 μl XF Base medium pH 7.4 (NaOH), containing 2 mM l-glutamine, with or without 10 μg/ml of doxycycline. After 1 hour of humidified incubation at 37 °C, glycolysis was evaluated on the XFp platform with the Seahorse XFp Glycolysis Stress Test Kit (Agilent Technologies). After three measurements under basal conditions, glucose was injected to a final concentration of 10 mM followed by three measurements. Then, oligomycin A was injected to a final concentration of 1 μM followed by three measurements. To finish, 2-deoxy-d-glucose was injected to a final concentration of 50 mM followed by three measurements.

On each XFp cell culture microplate, triplicates of doxycycline-treated cells were compared with triplicates of vehicle treated cells. To determine the amount of protein, the wells were aspirated, rinsed with PBS and cells were lysed with 1% Triton X-100 in PBS, followed by a protein assay with the Pierce BCA Protein Assay Kit. All experiments were repeated 5–6 times. Data were analysed with Wave Desktop 2.6 software and a Microsoft Excel Macro provided by Agilent Technologies. Results were expressed per amount of protein.

### Determination of fraction of cells in S-phase and dead cells

Cells were seeded at low density on polylysine-coated glass coverslips and cultured in 6-well plates. From the day after seeding, the cells were treated daily with 10 μg/ml of doxycycline or vehicle in fresh medium over a 5-day period. During the last 4 hours of day 5, the cell culture medium was supplemented with BrdU to a final concentration of 15 μM (5-Bromo-2′-deoxy-uridine Labeling and Detection Kit I, Sigma-Aldrich). Incorporated BrdU was detected immunocytochemically with the anti-BrdU antibody from Kit I and Alexa Fluor 488-labeled goat anti-mouse IgG (Abcam) secondary antibody according to the kit’s manual. Coverslips were mounted on microscope slides in Citifluor/Glycerol/PBS solution AF1 (Agar Scientific) supplemented with of 1 μg/ml of 4′,6-diamidino-2-phenylindole (DAPI; Sigma-Aldrich). Coverslips were examined under a Zeiss Axiophot microscope. Random images were captured with a Zeiss Axiocam MRm camera and were processed with Adobe Photoshop software. Per coverslip, >250 cells, visualized by DAPI fluorescent blue nuclear counterstaining, were counted and the number of cells showing BrdU incorporation (green fluorescent antibody staining) was determined. Experiments were carried out in quadruplicate.

To determine the fraction of dead cells relative to the total cell number, non-adhered and adhered cells were collected, resuspended in PBS containing 0.5 μg/ml of propidium iodide (Sigma-Aldrich) and analysed with the PI Viability assay on a Moxi GO flow cytometer (Orflo Technologies) in quadruplicate.

### Caspase activity assays

To examine the effect of doxycycline with or without gemcitabine on caspase 9, 8 and 3/7 activities, 7.0 × 10^4^ fibroblasts, 1.0 × 10^4^ A549 cells or 3.0 × 10^4^ A549 ρ° cells were seeded on four 10-cm tissue culture dishes each. From the day after seeding, two plates of each culture were treated daily with 10 μg/ml of doxycycline in fresh medium and two plates were treated daily with vehicle in fresh medium over a 5-day period. During the last 2 days of treatment, of each group of two plates one plate was also treated with 10 ng/ml (33 nM) of gemcitabine (Supplementary Fig. S1). Gemcitabine (2′,2′-difluoro-2′-deoxycytidine) was added to fresh culture medium from a 10 μg/ml stock of gemcitabine hydrochloride (Sigma-Aldrich) in water. After 5 days of treatment, cells were dislodged by trypsinisation and a suspension of 20,000 cells in 100 μl of culture medium with or without the drugs were transferred to multiple wells of a Bio-One μClear white 96-well plate and moved to a cell culture incubator. Culture medium was used as blank. After 1 hour, the 96-well plate was equilibrated to room temperature and 100 μl of Caspase-Glow 9, Caspase-Glow 8 or Caspase-Glow 3/7 reagent (Promega), prepared according to the manufacturer’s manual, was added to each well. The plate was covered with plate sealer and mixed on a plate shaker at 300 rpm for 30 seconds, followed by 1 hour incubation at room temperature. The luminescent signal was recorded with a Fluoroskan FL plate reader. The background value (blank wells) was subtracted from the experimental values. Experiments were performed in quadruplicate.

### ATP assays

Logarithmically growing cell cultures were treated daily with 10 μg/ml of doxycycline or vehicle in fresh medium. After 4 days of treatment, 8 × 10^3^ cells of each culture were replated in multiple wells of Bio-One μClear black 96-well plates and cultured with 10 μg/ml of doxycycline or vehicle in fresh medium. The next day, the medium in the wells was replaced with 100 μl of culture medium containing either 25 mM d-(+)-glucose (Sigma-Aldrich) or 25 mM 2-deoxy-d-glucose (Sigma-Aldrich). After 35 minutes of culturing, the medium in half of the glucose containing wells was replaced with 100 μl of medium containing 25 mM of glucose, 2 μM oligomycin A (Sigma-Aldrich), and the medium in half of the 2-deoxyglucose containing wells with 100 μl of medium containing 25 mM 2-deoxyglucose, 2 mM oligomycin A. After 25 minutes of culturing, an empty row on the 96-well plate was filled with 100 μl of medium and 10 μl of a 0–20 μM ATP dilution series. Then, 50 μl of mammalian cell lysis solution from the ATPlite Luminescence ATP Detection Assay System (Perkin Elmer) was added to each well, plates were covered with plate sealer and mixed on a plate shaker at 450 rpm for 5 minutes. Next, 50 μl of reconstituted substrate solution from the ATPlite Assay System was added to each well and plates were shaken again at 450 rpm for 5 minutes. Plates were kept in the dark for 5 minutes prior to recording of the luminescent signal with a BioTek Synergy HT plate reader. After reading of the luminescence, the cell number in each well was determined with the CyQUANT Cell Proliferation Assay Kit (Thermo Fisher Scientific). An empty row on the 96-well plates was filled with 100 μl of a serial dilution of 0–25,000 cells in culture medium, 50 μl of lysis solution and 50 μl of substrate solution from the ATPlite Assay System, and mixed. Then, 100 µl of CyQUANT GR dye/cell lysis solution, prepared as directed by the manufacturer’s protocol, was added to each well and mixed. The fluorescent signal was recorded at 480 mm excitation and 520 nm emission with a Synergy HT plate reader. Standard curves were constructed for amount of ATP *versus* the luminescent signal and the number of cells *versus* the fluorescent signal in Microsoft Excel. Equations of the standard curves were used to calculate the amount of ATP in 1000 cells. Experiments were performed in quadruplicate.

### Assessment of ΔΨ_m_ and mitochondrial mass

Cells were seeded at low density in 35-mm μ-dishes with a glass bottom (Ibidi). From the day after seeding, cells were treated daily with 10 μg/ml of doxycycline or vehicle in fresh medium. After 5 days of treatment, cells were rinsed with Hanks’ balanced salt solution (HBSS; Gibco Lifetechnologies), followed by incubation in 1 ml of 200 nM MitoTracker Green FM (Thermo Fisher Scientific), 25 nM TMRM (Thermo Fisher Scientific) in HBBS with or without 10 μg/ml of doxycycline. After 15 minutes, the solution was replaced by 25 nM TMRM in HBSS. The fluorescent signals of MitoTracker Green FM (green) and TMRM (red) in the cells was recorded with a Nikon Eclipse Ti-E inverted confocal laser-scanning microscope, equipped with a ×60 objective, 15 minutes after the second addition of TMRM, when this dye was fully equilibrated. After this measurement, 0.5 ml of 12 μM carbonyl cyanide 3-chlorophenylhydrazone (CCCP; Sigma-Aldrich) in HBSS was added to dissipate ΔΨ_m_ and the fluorescence was recorded for 2 minutes. Imaging data were collected with NIS-Elements software (Nikon). For the measurements, 7 z-stacks of 0.1 μm were projected. Single cells were selected as regions of interest and ‘area’, ‘mean grey value’ and ‘integrated density’ were calculated with Image J software (National Institutes of Health) and used to calculate the ‘background-corrected total cell TMRM fluorescence’ (*i.e*. integrated density - [area of selected cell × mean fluorescence of background readings]). The MitoTracker Green FM signal was binarised to determine the mitochondrial footprint per cell and used to express the TMRM fluorescence intensity per mitochondrial content per cell. In separate experiments, MitoTracker Green FM fluorescence per cell was used as measure of mitochondrial mass. Per dish, 12–47 cells were analysed. Experiments were carried out in triplicate.

### Measurement of cytosolic ROS production rate

Cells were seeded at low density in μ-dishes and treated with doxycycline or vehicle as described above for the ΔΨ_m_ experiments. After 5 days of treatment, cells were rinsed with HBSS, followed by incubation in 1 ml of 10 μM DHE (Thermo Fisher Scientific) in HBBS with or without 10 μg/ml of doxycycline. After 10 minutes of incubation, the fluorescent signal in the cells was recorded with a Nikon Eclipse Ti-E microscope as described above over a 10-minute period. To analyse the increase in nuclear staining intensity over the 10-minute period, 7 z-stacks of 0.1 μm were projected, single nuclei were selected as regions of interest and the background-corrected total nuclear DHE fluorescence was determined at the start of the recording and 10 minutes later. The nuclear signal was binarised to create a mask, and used to express the increase in fluorescence per nucleus per minute. Per dish, 14–46 cells were analysed. Experiments were carried out in quadruplicate.

### Assessment of cellular GSH levels

Cells were seeded at low density in μ-dishes and treated with doxycycline or vehicle as described above for the ΔΨ_m_ experiments. After 5 days of treatment, cells were rinsed with HBSS, followed by incubation in 1 ml of 50 μM mBCI (Thermo Fisher Scientific), 1 mM calcein AM (Thermo Fisher Scientific) in HBBS with or without 10 μg/ml of doxycycline. After 30 minutes of incubation, the fluorescent signals of calcein (green) and mBCI (blue) in the cells were recorded with a Nikon Eclipse Ti-E microscope as described above. For the measurements, 7 z-stacks of 0.1 μm were projected. Single cells were selected as regions of interest and the background-corrected total cell mBCI fluorescence was determined. The calcein cell signal was binarised to create a mask and used to express the mBCI fluorescence per cell area. Per dish, 11–28 cells were analysed. Experiments were carried out in triplicate. To verify that the mBCI fluorescent signal reflects the level of GSH, A549 cells were cultured for 5 days with or without 250 μM of the γ-glutamylcysteine inhibitor l-buthionine-sulfoximine (Sigma-Aldrich)^64^, followed by cellular mBCI fluorescence measurements. The treatment with l-buthionine-sulfoximine resulted in noticeably less blue fluorescent staining compared to untreated cells.

### Statistical analyses

Graphs and statistical analyses were executed with GraphPad Prism version 6.01 software. Data are presented as mean±standard deviation. As the sample size in most experiments was too small to confirm normal distribution, we used non-parametric Kruskal-Wallis tests to examine statistical significance. Mann-Whitney tests were used for pairwise comparisons. Statistical significance levels were set to p < 0.05 with Bonferroni correction for multiple pairwise comparisons.

### Data availability

The datasets generated and analysed during the current study are available from the corresponding author on reasonable request.

## Supplementary Information

Supplementary Information.
