# Supplementary material for: Mitochondria as target to inhibit proliferation and induce apoptosis of cancer cells: the effects of doxycycline and gemcitabine
Source: Sci Rep. 2020 Mar 9;10:4363. doi: 10.1038/s41598-020-61381-9 (PMC7063048; doi:10.1038/s41598-020-61381-9)
Supplement: Supplementary file 1 — Supplementary Information. [file 41598_2020_61381_MOESM1_ESM.pdf]

Supplementary information for:

Mitochondria as target to inhibit proliferation and induce apoptosis of cancer cells: the effects of doxycycline and gemcitabine

Sas N. Dijk[1], Margherita Protasoni[2], Marilena Elpidorou[2], Albert M. Kroon[2] and Jan-Willem Taanman[2,*]

[1]Research Department of Surgical Biotechnology, Division of Surgery and Interventional Science, University College London, London, NW3 2PF, UK
[2]Department of Clinical and Movement Neurosciences, Queen Square Institute of Neurology, University College London, London, NW3 2PF, UK

*Correspondence: Dr J.-W. Taanman, Department of Clinical and Movement Neurosciences, Queen Square Institute of Neurology, University College London, Royal Free Campus, Rowland Hill Street, London NW3 2PF, United Kingdom, Phone: +44-20-8016 8148, E-mail: j.taanman@ucl.ac.uk

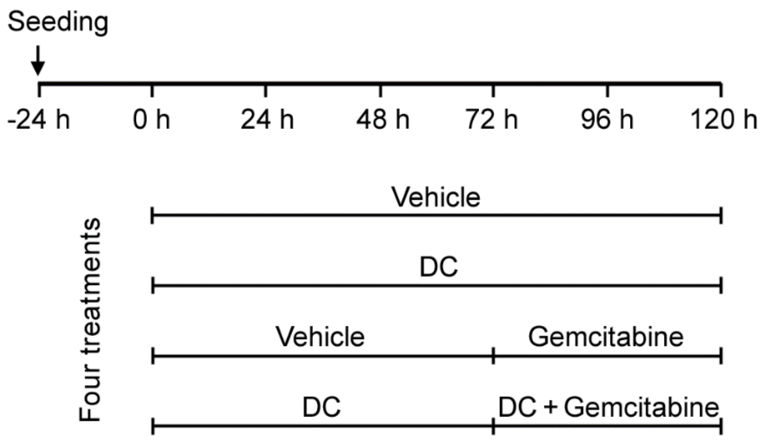

Supplementary Figure S1. Cell culturing and treatment scheme. Medium was changed every 24 h. DC, doxycycline.

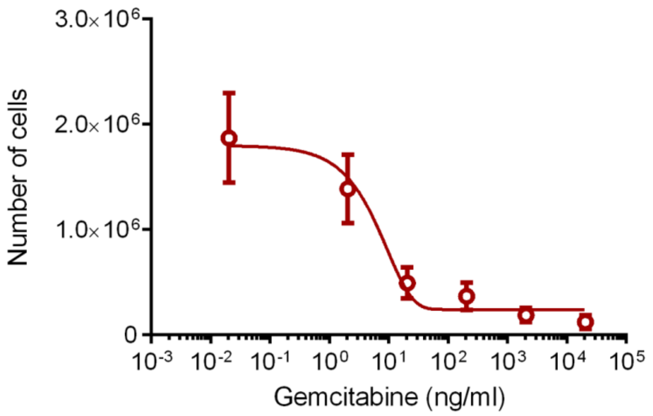

Supplementary Figure S2. Dose response curve for treatment of A549 cells with gemcitabine. To construct the dose-response curve, 1.0 × 10^4 A549 cells were seeded on 10-cm tissue culture dishes at t=-24 h. At t=72 h and t=96 h cells were treated with gemcitabine in fresh medium. Cells were counted at t=120 h (n=3). Symbols show mean number of cells. Error bars indicate standard deviations.

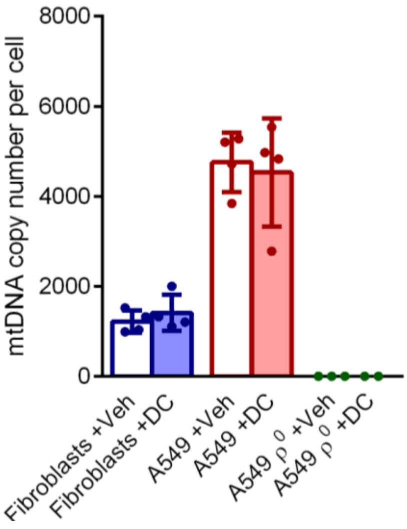

Supplementary Figure S3. Doxycycline has no effect on mtDNA copy number. Mean mtDNA copy number per cell after the cultures had been treated for 5 days with vehicle (Veh) or doxycycline (DC) (n=4). Error bars indicate standard deviations.

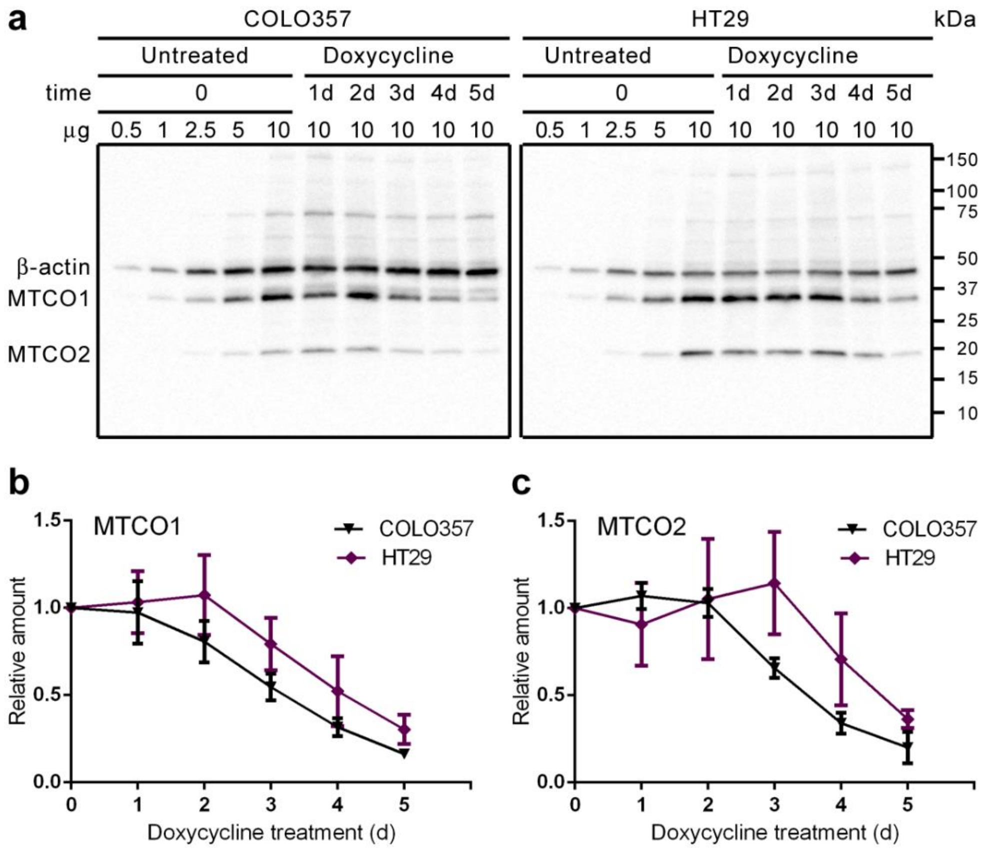

Supplementary Figure S4. Doxycycline decreases mitochondrial-encoded protein levels in COLO357 and HT29 cells. (**a**) Western blot images of samples from COLO357 and HT29 cells treated with doxycycline over a 5-day time period. To facilitate quantification, serial dilutions of untreated cells, harvested at t=0, were also applied. Blots were probed with antibodies against the mitochondrial-encoded proteins MTCO1 and MTCO2, and β-actin. Migration of protein standards is indicated on the right. (**b, c**) Mean amounts of MTCO1 and MTCO2 in the treated cells over a 5-day period relative to the amount in the cells at t=0 (n=3). Error bars indicate standard deviations.

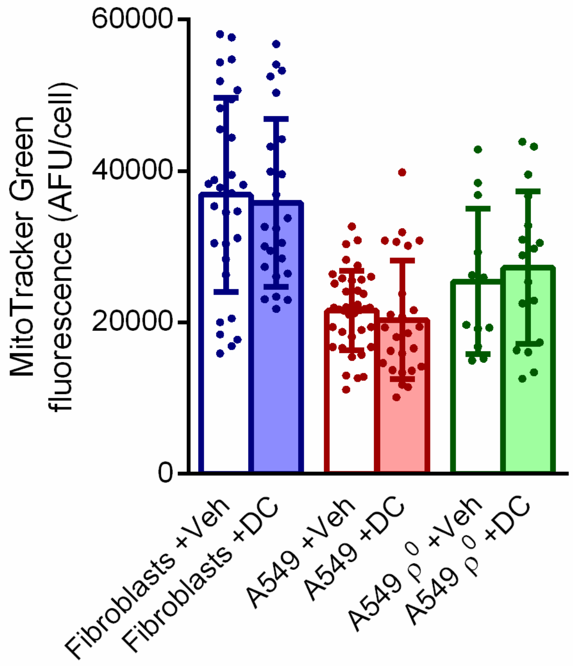

Supplementary Figure S5. Doxycycline has no effect on mitochondrial mass. Mean MitoTracker Green fluorescence per cell (n≥12) in arbitrary fluorescent units (AFU). Error bars indicate standard deviations

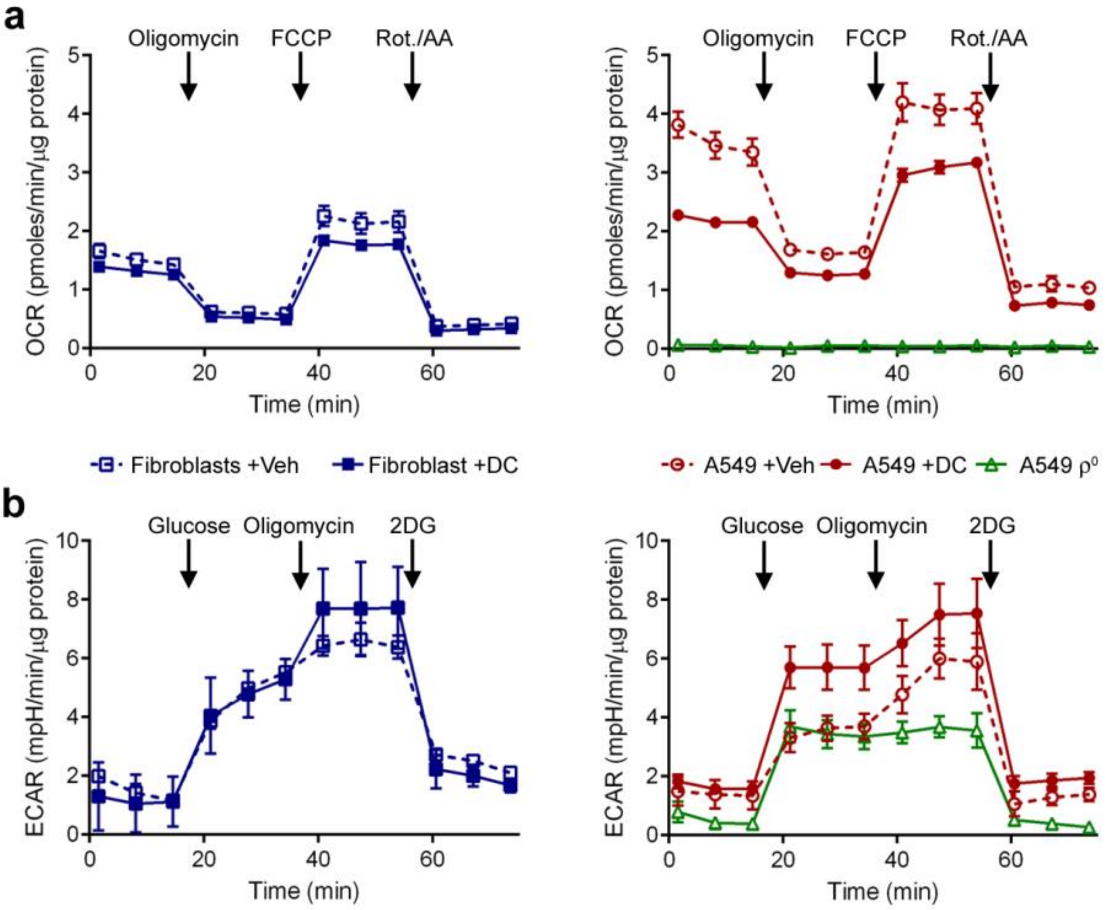

Supplementary Figure S6. Mitochondrial and glycolysis stress test profiles. (**a**) Oxygen consumption rates (OCR) of representative mitochondrial stress tests of fibroblasts and A549 cells treated with vehicle (Veh) or doxycycline (DC) for 5 days, and A549 ρ^0 cells. Oligomycin A, FCCP and rotenone/antimycin A (Rot./AA) were added sequentially to dissect oxidative phosphorylation function. (**b**) Extracellular acidification rates (ECAR) of representative glycolysis stress tests of fibroblasts and A549 cells treated with vehicle or doxycycline for 5 days, and A549 ρ^0 cells. Glucose, oligomycin A and 2-deoxyglucose (2DG) were added sequentially to dissect glycolytic function. Symbols represent mean values. Error bars indicate standard deviations of 3 technical repeats.

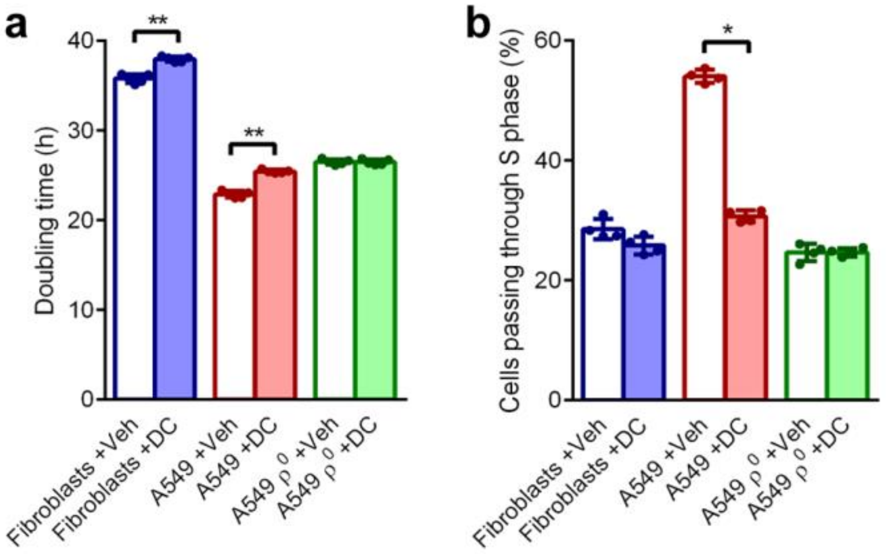

Supplementary Figure S7. Doxycycline decreases cellular proliferation. (**a**) Mean doubling time of cells over 5 days of culturing with vehicle (Veh) or doxycycline (DC) (n=5). (**b**) Mean percentage of cells passing through S-phase after the cultures had been treated for 5 days with vehicle or doxycycline (n=4). Error bars indicate standard deviations. Asterisks denote statistically significant differences (*p<0.05; **p<0. 01).

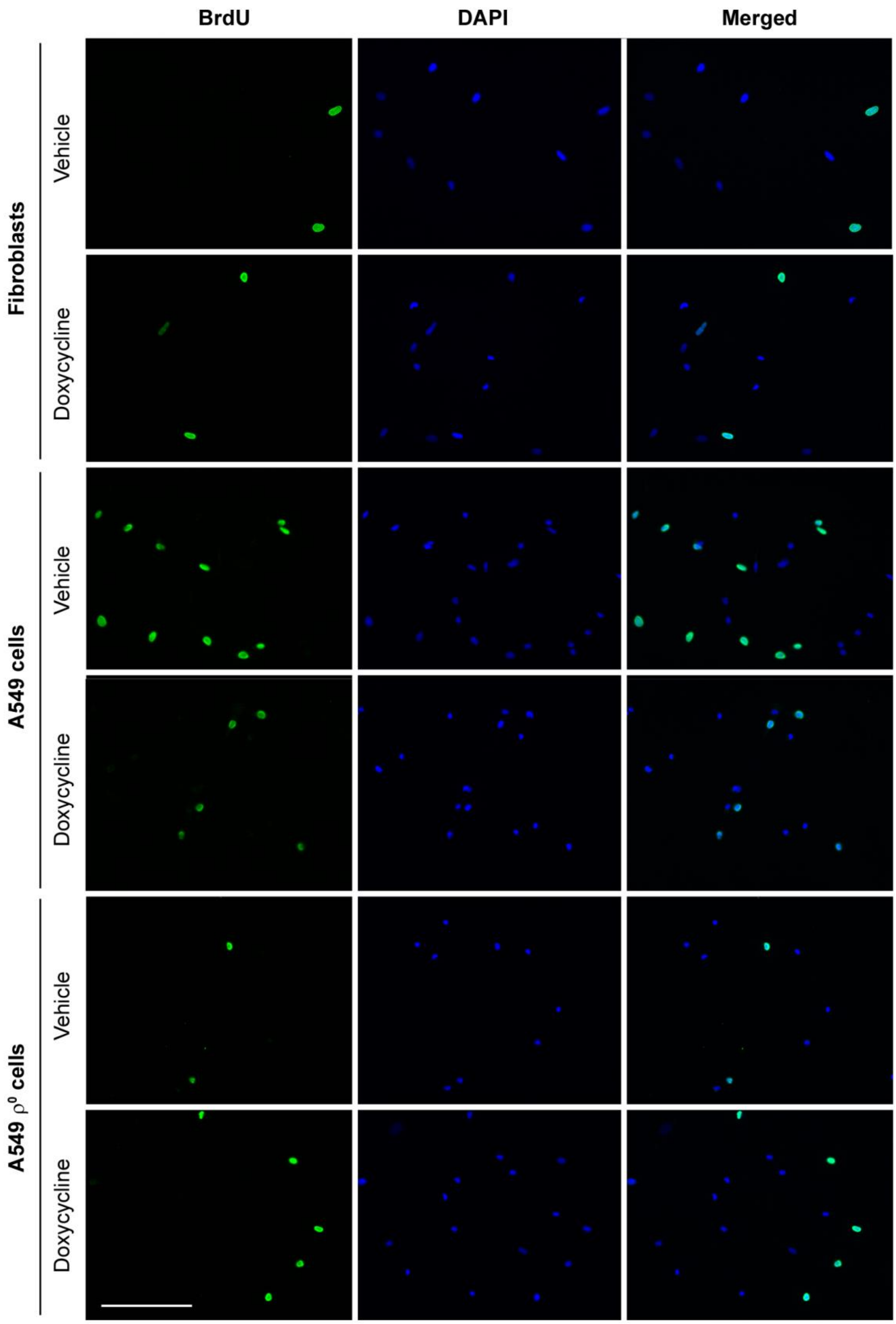

Supplementary Figure S8. Representative fluorescent micrographs of BrdU incorporation into replicating nuclear DNA. Fibroblast, A549 and A549 ρ^0 cultures were treated with vehicle or doxycycline for 5 days and with BrdU during the last 4 hours, followed by immunocytochemical staining of BrdU incorporation (green) and DAPI nuclear counterstaining (blue). Scale bar: 100 μm.

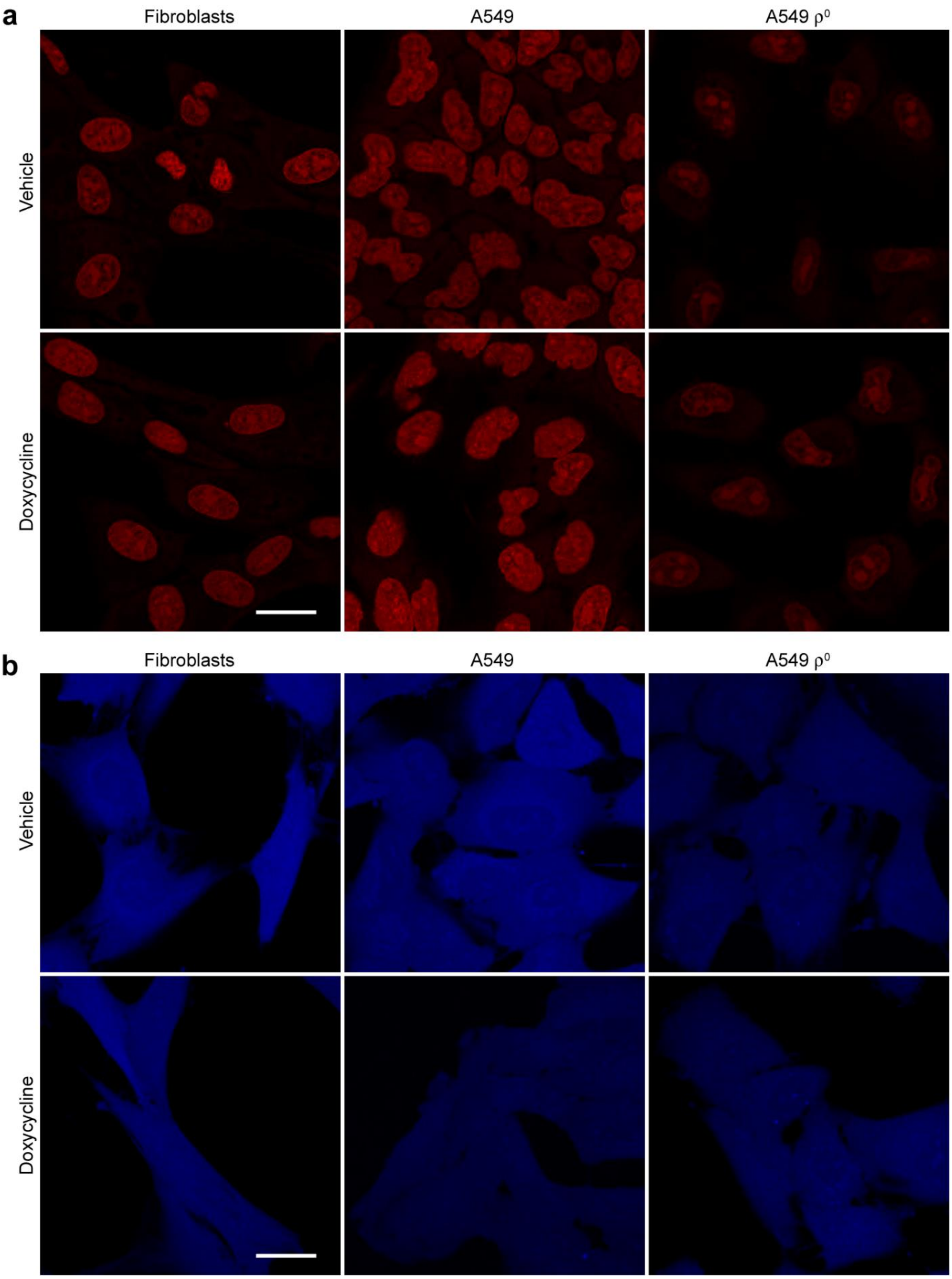

Supplementary Figure S9. (**a**) Representative fluorescent micrographs of DHE staining. Fibroblast, A549 and A549 ρ^0 cultures were treated with vehicle or doxycycline for 5 days, followed by staining with the ROS indicator dye DHE. (**b**) Representative fluorescent micrographs of mBCI staining. Fibroblast, A549 and A549 ρ^0 cultures were treated with vehicle or doxycycline for 5 days, followed by staining with the GSH indicator dye mBCI. Scale bars: 10 μm.

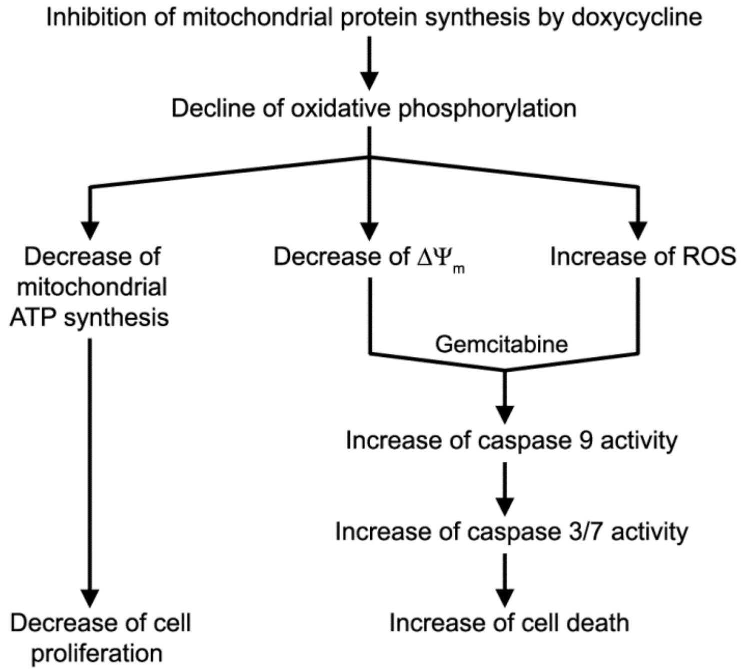

Supplementary Figure S10. Proposed impact of doxycycline and gemcitabine on cellular physiology and apoptosis. Doxycycline causes inhibition of mitochondrial protein synthesis. This impedes mitochondrial oxidative phosphorylation, which in turn leads to a decrease of mitochondrial ATP synthesis and $\Delta\Psi_m$, and an increase of ROS. These physiological changes reduce the cellular proliferation rate and lower the apoptotic threshold. When the cell is subsequently insulted by gemcitabine, caspase 9 is activated and activates caspase 3/7, resulting in apoptosis.

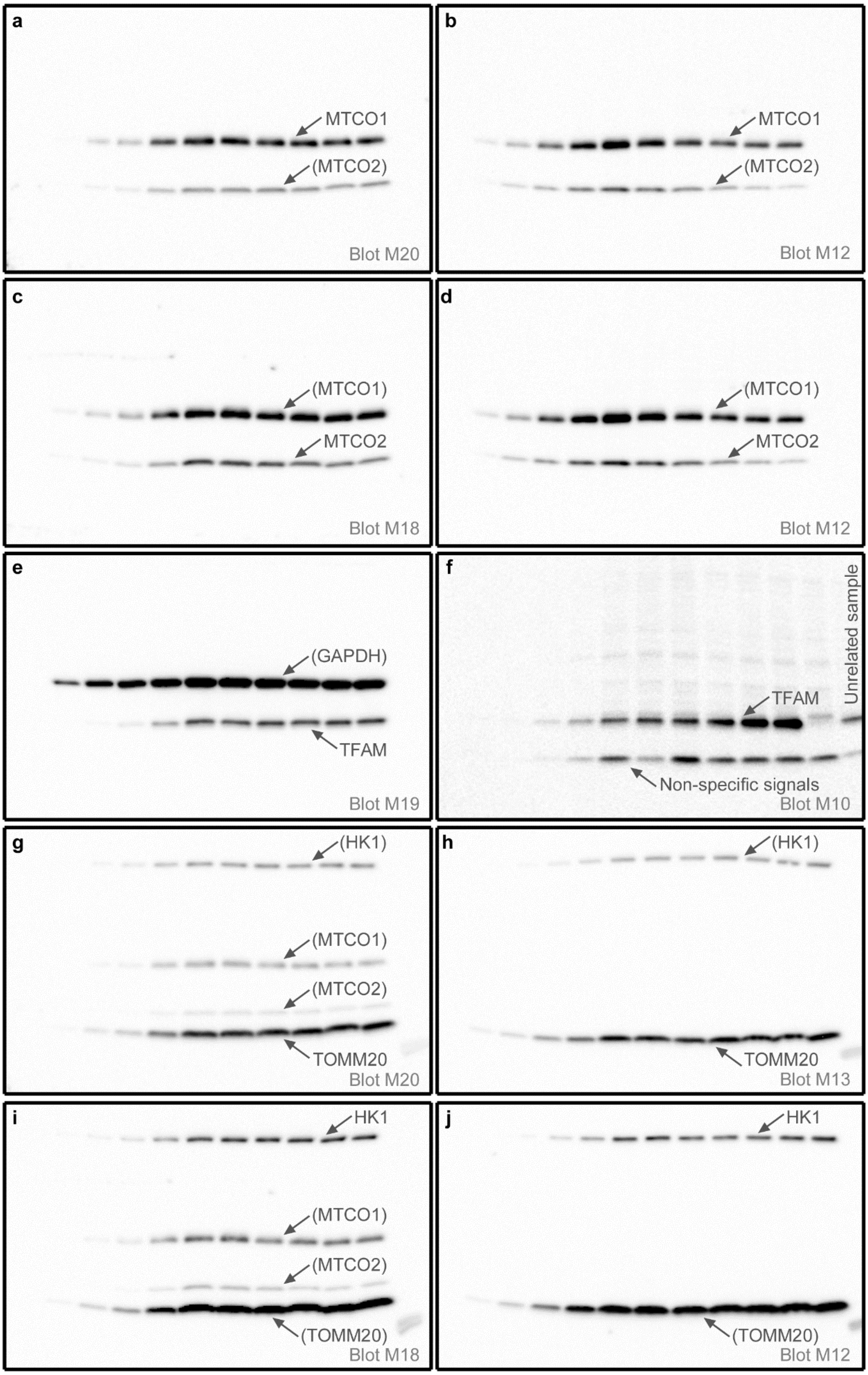

a
MTCO1
(MTCO2)
Blot M20
b
MTCO1
(MTCO2)
Blot M12
c
(MTCO1)
MTCO2
Blot M18
d
(MTCO1)
MTCO2
Blot M12
e
(GAPDH)
TFAM
Blot M19
f
Unrelated sample
TFAM
Non-specific signals
Blot M10
g
(HK1)
(MTCO1)
(MTCO2)
TOMM20
Blot M20
h
(HK1)
TOMM20
Blot M13
i
HK1
(MTCO1)
(MTCO2)
(TOMM20)
Blot M18
j
HK1
(TOMM20)
Blot M12

..

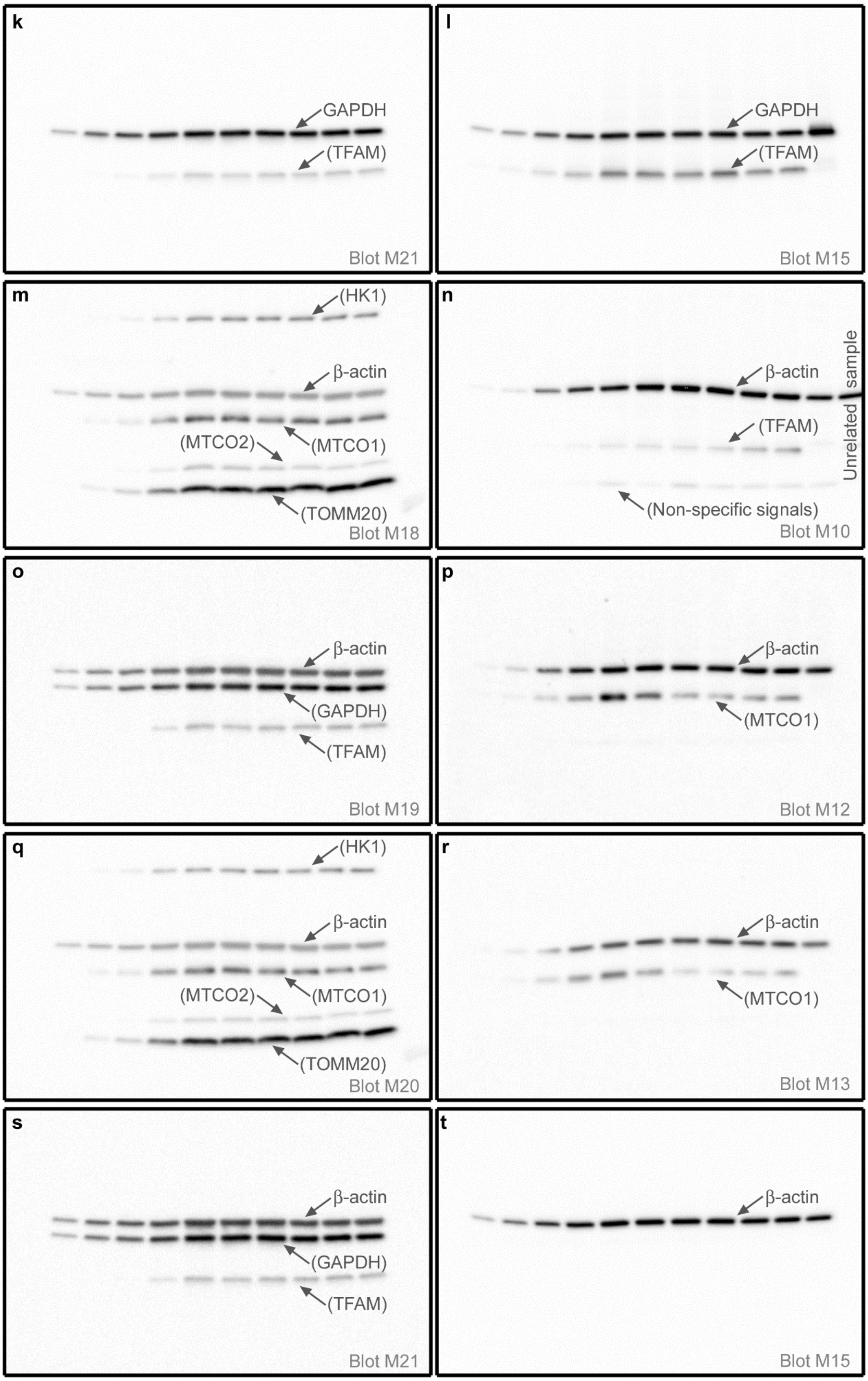
k
GAPDH
(TFAM)
Blot M21
l
GAPDH
(TFAM)
Blot M15
m
(HK1)
β-actin
(MTCO2)
(MTCO1)
(TOMM20)
Blot M18
n
β-actin
(TFAM)
Unrelated sample
(Non-specific signals)
Blot M10
o
β-actin
(GAPDH)
(TFAM)
Blot M19
p
β-actin
(MTCO1)
Blot M12
q
(HK1)
β-actin
(MTCO2)
(MTCO1)
(TOMM20)
Blot M20
r
β-actin
(MTCO1)
Blot M13
s
β-actin
(GAPDH)
(TFAM)
Blot M21
t
β-actin
Blot M15

Supplementary Figure S11. Full-size of blots shown in Fig. 1c at exposure times that gave non-saturated signals used for quantification. Protein symbols in brackets indicate signals of co-probing or earlier probing. (**a**, **c**, **e**, **g**, **i**, **k**, **m**, **o**, **q**, **s**) Blots loaded with fibroblast samples and probed with (**a**) anti-MTCO1 antibodies, (**c**) anti-MTCO2 antibodies, (**e**) anti-TFAM antibodies, (**g**) anti-TOMM20 antibodies, (**i**) anti-HK1 antibodies, (**k**) anti-GAPDH antibodies and (**m**, **o**, **q**, **s**) anti-β-actin antibodies. (**b**, **d**, **f**, **h**, **j**, **l**, **n**, **p**, **r**, **t**) Blots loaded with A549 cell line samples and probed with (**b**) anti-MTCO1 antibodies, (**d**) anti-MTCO2 antibodies, (**f**) anti-TFAM antibodies, (**h**) anti-TOMM20 antibodies, (**j**) anti-HK1 antibodies, (**l**) anti-GAPDH antibodies and (**n**, **p**, **r**, **t**) anti-β-actin antibodies.

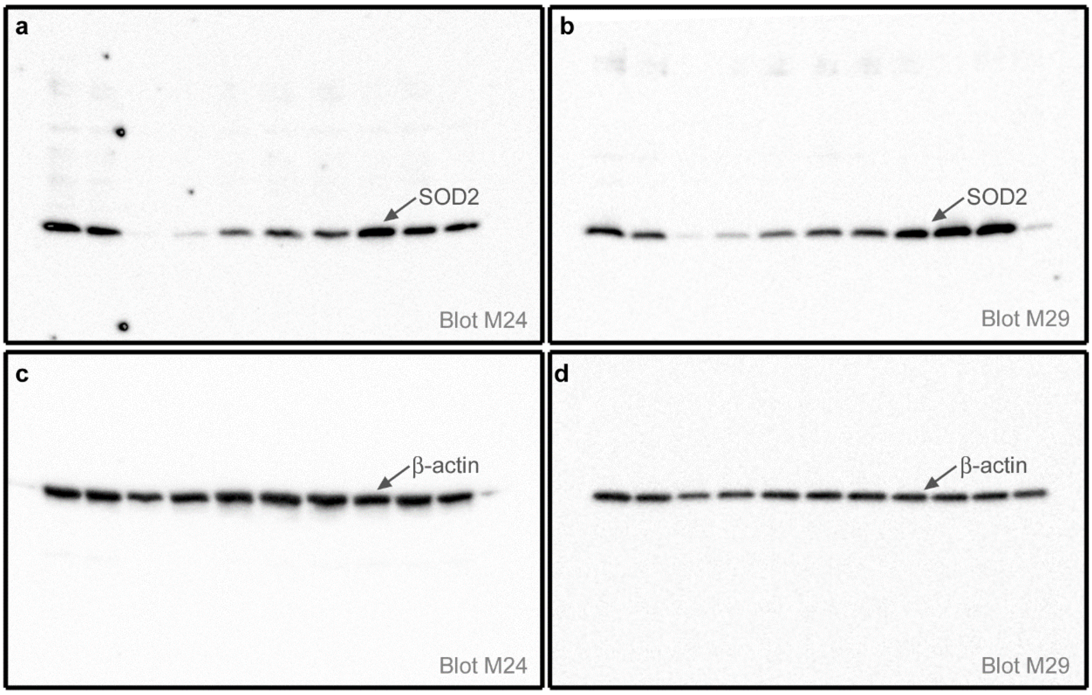

Supplementary Figure S12. Full-size of blots shown in Fig. 6a at exposure times that gave non-saturated signals used for quantification. (**a**, **c**) Blots loaded with fibroblast samples and probed with (**a**) anti-SOD2 antibodies and (**c**) anti-β-actin antibodies. (**b**, **d**) Blots loaded with A549 cell line samples and probed with (**b**) anti-SOD2 antibodies and (**d**) anti-β-actin antibodies.
